# Toward precision medicine of breast cancer

**DOI:** 10.1186/s12976-016-0035-4

**Published:** 2016-02-29

**Authors:** Nicolas Carels, Lizânia Borges Spinassé, Tatiana Martins Tilli, Jack Adam Tuszynski

**Affiliations:** Laboratório de Modelagem de Sistemas Biológicos, National Institute of Science and Technology for Innovation in Neglected Diseases (INCT/IDN, CNPq), Centro de Desenvolvimento Tecnológico em Saúde, Fundação Oswaldo Cruz, Rio de Janeiro, Brazil; Department of Oncology, Faculty of Medicine & Dentistry, University of Alberta, Edmonton, AB T6G 1Z2 Canada; Department of Physics, University of Alberta, Edmonton, AB T6G 2E1 Canada

**Keywords:** Hallmarks, Omics, Profiling, Therapy, Stem cell, Signaling networks, Tumor heterogeneity, Pharmacogenetics

## Abstract

In this review, we report on breast cancer’s molecular features and on how high throughput technologies are helping in understanding the dynamics of tumorigenesis and cancer progression with the aim of developing precision medicine methods. We first address the current state of the art in breast cancer therapies and challenges in order to progress towards its cure. Then, we show how the interaction of high-throughput technologies with *in silico* modeling has led to set up useful inferences for promising strategies of target-specific therapies with low secondary effect incidence for patients. Finally, we discuss the challenge of pharmacogenetics in the clinical practice of cancer therapy. All these issues are explored within the context of precision medicine.

## Background

Breast cancer (BC) is a global disease; it is the most common cancer in women (accounting for 25 % of all cancers), with nearly 281,840 estimated new cases, and 40,290 estimated deaths in 2015 in the US population (http://seer.cancer.gov), which account for ~320 million people. BC is also becoming an increasingly urgent problem in low- and middle-income countries, such as Brazil where government estimates BC as the major malignant neoplasia in women and the main cause of death from cancer in the country. This fact has been associated with increased life expectancy, urbanization, and high-risk cancer-causing behaviors such as tobacco smoking [1].

The shortcomings of *one-size-fits-all* approach (an approach that is standard and not tailored to individual needs) to treatments are well reflected in the disappointing outcome of current chemotherapies, where drug agents directed at an individual target often show limited efficacy and safety due to factors such as off-target side effects, bypass mechanisms and cross-talk across compensatory escape pathways [2] due to genome destabilization and signaling rewiring. Because, malignant rewiring is induced by apparently random genomic perturbation, therapy improvement has to go through *precision medicine* (PM).

By contrast to *stratified medicine* (SM), which consists in indicating a drug for a population according to a specific molecular alteration, PM aims to indicate a treatment individually [3]. Thus, PM is a medical model that proposes the customization of healthcare, with medical decisions, practices, and/or products being tailored to the individual patient. In this model, diagnostic testing is often employed for selecting appropriate and optimal therapies based on the context of a patient’s genetic profile or other molecular or cellular analysis [4, 5]. At the moment SM is the dominant model; it is divided into two different types of molecular screening programs: basket trials and umbrella trials. The basket trials test the effect of a single drug on a molecular alteration in a variety of cancers while the umbrella trials assess the effect of different drugs in different molecular alterations either in one or several tumours [3].

Despite still disappointing results partly due to incorrect or imprecise prevailing views and technology limitations, PM remains an indispensable route to decrease the toxicity of cancer treatment and to increase its benefit to patients. A mutation-oriented approach is not expected to solve cancer therapy because if genome destabilization is effectively due to these mutations, cellular dysregulation results to a greater degree from genome destabilization than from such mutations. Recent progresses in high throughput generation of genome, transcriptome, proteome, and interactome data as well as *in silico* data mining offer the possibility of unprecedented high precision diagnosis at prices that become affordable. The integration of sciences through informatics and mathematical modeling constitutes a new opportunity to improve cancer therapies through PM. Thus, it is the aim of this report to review the traditional approach that is given to BC treatment and the benefit that breakthrough technologies, modeling and data manipulations may provide to traditional limitations in the prospective of PM applied to BC.

## Review

### Incidence of breast cancer and prevention

Cancer incidence varies among countries mainly according to lifestyle, which explains as much as 75-85 % of cancer etiology, with the most significant parameters being: physical inactivity, obesity, extensive working hours, intensive exposure to carcinogens, hormonal contraceptive use, postmenopausal hormone replacement therapy, nulliparity, late age at first birth, and enhanced alcohol consumption [6]. Lifestyle’s influence on cancer likelihood has been demonstrated by statistics from people migrating from their native country to an adopted country starting to mimic the risk profile and cancer incidence of their adopted country (especially USA). For instance, populations consuming high levels of plant derived foods have low incidence rates of various cancers particularly in Southern European (Mediterranean countries) compared to Northern European countries. Similarly, populations in South East Asian countries have a much lower risk of developing numerous cancers compared to their more industrialized, Western counterparts [7]. Countries with lower cancer incidence were associated with a nutrition mostly based on vegetables, fruits and fishes rather than on red meat and animal fats. The compounds that have been most cited as being cancer protective include those that belong to phenolics comprised of at least 8,000 chemical species throughout the plant kingdom with their main representative belonging to shikimic acid, phenylpropanoid and flavonoid biosynthetic pathways [8–10]. The main action of these compounds is to prevent cancer development by promoting anti-oxidant and anti-inflammatory effects as well as inducing cell cycle arrest, cell survival and apoptosis or programmed cell death [7]. Because of the pleiotropic effect of plant compounds, the exact contribution of a diet based on plant products to cancer prevention is difficult to unwrap [11]. Examples of plant compounds used in cancer therapy are: curcumin, genistein, resveratrol and catechins.

## Mammary gland complexity and cell type diagnosis

The mammary gland is a complex organ constituted by two tissue compartments, i.e. epithelium and stroma, which undergoes cycles of proliferation, differentiation and apoptosis in response to local and endocrine signals. It is the highly dynamic epithelium that undergoes major functional differentiation upon pregnancy to produce milk in response to local and endocrine signals. The epithelium of the mammary gland is made of luminal and basal/myoepithelial cells. Luminal cells line the ductal lumen and secrete milk upon terminal differentiation into lobulo-alveolar cells while basal/myoepithelial cells are lodged just below luminal cells and ensure ductal contractility to release milk [12]. Breast duct are also infiltrated with stem cells (SC) tightly regulated to produce all cellular elements that make up breast ducts and, therefore, play a critical role in normal gland development and cycling. SCs normally undergo asymmetric division to generate a copy of the original cell and a progenitor one that will suffer differentiation [13].

Stroma is a connective tissue whose main constituents, from a BC prospective, are adipocytes, fibroblasts, and endothelial cells; it is the mammary fat pad that supports the extensive system of ducts and alveoli. The functional mammary gland results from a succession of distinct stages under steroid and peptide hormonal control: (i) cyclical production of ovarian estrogen and progesterone accelerates ductal growth and branching during puberty, (ii) prolactin and placental lactogens control the proliferation and maturation of the alveolar compartment during pregnancy, and (iii) systemic concentration of prolactin and growth hormone decline with the increased pressure resulting from cessation of milk removal as well as loss of suckling stimuli [14].

**Fig. 1:**
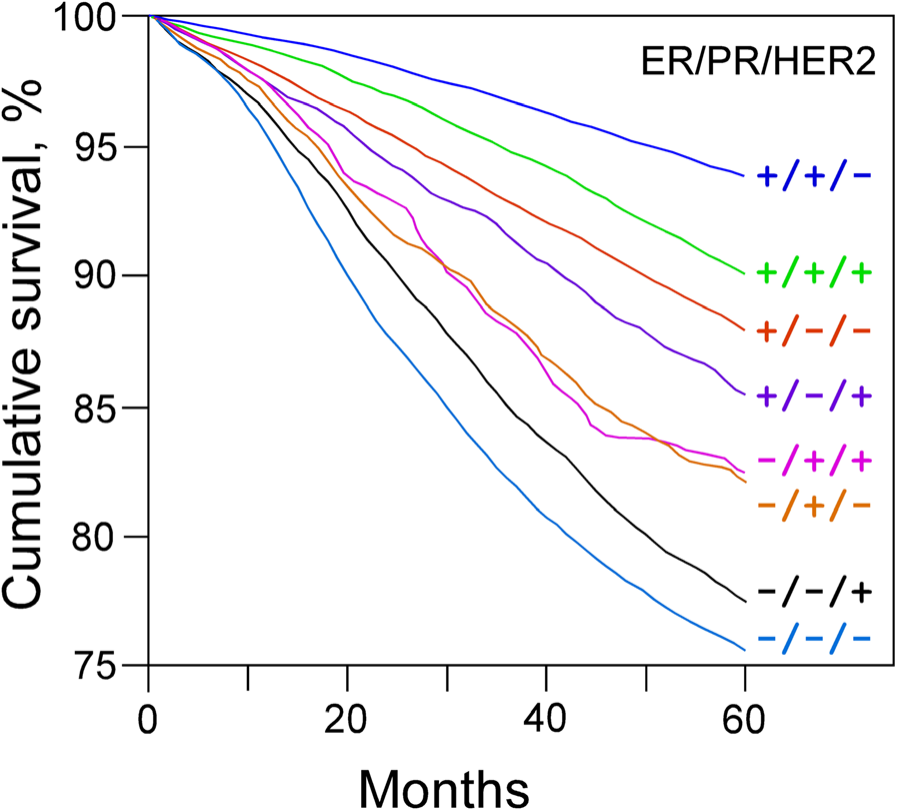
Kaplan-Meier graph illustrating the relative patient 5-years survival by tumor type according to time after treatment (modified from [17])

Following mammogram diagnosis, BC is usually classified primarily by its histological appearance (Table 1). Most BCs are derived from the epithelium compartment and are considered malignant according to their differentiation grade, which can be differentiated (low grade), moderately differentiated (intermediate grade), and poorly differentiated (high grade) as the cells progressively lose the features seen in normal breast cells. Poorly differentiated cancers have the worse prognosis. BC cells have receptors on their surface, in their cytoplasm and nucleus that can be used for molecular classification by histopathology and simple immunohistochemical procedures. Three primarily investigated receptors are the estrogen receptor (ER), progesterone receptor (PR), and human *epidermal growth factor receptor 2* (HER2, also known as ERBB2) because their status informs the physician in regard to how to proceed with specific therapies. When cancer cells express estrogen receptors, they depend on estrogen for their growth, so they can be treated with antagonist drugs (e.g. tamoxifen) to block estrogen effects on ER signaling cascade, and generally have a better prognosis. The majority of cells co-express ER and PR, which means that cells expressing one or both receptors are hormone receptor-positive (HR+) cells [15]. HER2+ cancer cells respond to biological agents such as the monoclonal antibody trastuzumab used in combination with conventional chemotherapy [16]. Cells that do not express these three receptor types are called triple-negative (TN); the lack of addressable molecular targets in these tumors is challenging and no FDA approved TN-specific treatments are currently available. Although they frequently express receptors for other hormones, such as androgen and prolactin, cells with a luminal phenotype are rarely observed in basal-like BCs [17]. TN patients have the worst prognosis [18] (as shown in Fig. 1). A number of studies have demonstrated that TN can be subclassified into six subtypes [19]. Notably, the three main markers above have been shown to have a high negative predictive value, but a limited positive predictive value. Hence, the development of molecular tools with better predictive power for patient outcome and response to treatment has long been a subject of great interest in translational research.

**Table 1:** State of hormonal receptors in several cell lines used as molecular markers for breast cancer diagnosis

Cell line	Histological subtype	ER/PR^a^	HER2^b^	EGFR^c^	CK5-6^d^
MCF10A	Control	0	0-1+	2+	+
MCF-7	LA^e^	6	0-1+	1+	-
T-47D	LA	>0	2+	-	-
ZR-75-1	LA	3-4	2+	1+	-
BT-474	LB^f^	0/8	3+	1+	-
BT-20	TN^g^	0	0-1+	2+	-
MB-231	TN	0	0-1+	1+	-
MB-468	TN	0	0	3+	-

^a^ *ER/PR* Estrogen/Progesterone receptor, ^b^ *HER2* Human epidermal growth factor receptor; ^c^ *EGFR* epidermal growth factor, ^d^ *CK5-6* Cytokeratin 5/6; ^e^ *LA* luminal A, ^f^ *LB* Luminal B, ^g^ *TN* triple-negative

Solid tumors represent a heterogeneous environment regarding access to oxygen and nutrients; thus their growth depends on the physical location of their malignant cells relative to these factors under the prevailing conditions. As a model of solid tumors, floating sphere-forming assays (mammosphere) are broadly used to test SC activity in tissues, tumors and cell lines. Spheroids originate from a small population of cells with SC features, which are able to grow in a suspension culture and behaving tumorigenically in mice [20]. Because the classification of malignant cells is a key parameter for a successful development of a therapy, considerable efforts are being invested in molecular characterization of malignant cells. In particular, breast cancer stem cells (BCSC) generated substantial interest because they are thought to play the role of a common ancestor for most tumor cells. In that regard, markers for BCSC would allow the routine blood diagnosis diminishing the necessity for invasive biopsies [21]. Actually, none of the known markers are specific for BCSC, and only new cell surface marker combinations may improve the reliability, identification, and enrichment of BCSCs [22]. Clusters of differentiation (CD) are antigens expressed on cell surface that are used to diagnose cellular populations according to their type and function using specific antibodies. Today, more than 360 different CDs have been identified. The surface cell markers *epithelium cancer adhesive molecule* (Ep-CAM) and CD49f (α-6 integrin) were investigated in that context. It was found that the combination Ep-CAM^high^/CD49f^neg^ cells represent the differentiated luminal cells, while the combination Ep-CAM^-/low^/CD49f^+^ phenotype characterize mainly the basal fraction of the human epithelial cells [23]. However, it has also been shown that the majority of BC cells have a luminal Ep-CAM^high^/CD49f^+^ phenotype, and the identification of CD44^high^/CD24^low^ status significantly improves flow cytometry diagnosis of BC forming SCs [24]. Thus BCSC classification allowed to show that epithelial population of *basal A* progenitor cells (Ep-CAM^-/low^/CD49f^+^), *luminal B* progenitor cells (Ep-CAM^high^/CD49f^+^), and *luminal differentiated C* cells (Ep-CAM^high^/CD49f^−^) differ in their ability to form mammospheres and colonies in such a way that A > B while C does not possess these abilities [22, 24]. At the moment, the very low blood concentration of SC is a hurdle for liquid biopsy, but a new development in nanotechnology suggests that mechanical and optoplasmonic transduction will soon allow the detection of cancer biomarkers in serum at ultra-low concentrations such as ~10^−16^ g/ml [25].

## Carcinogenesis process and consequences for patients

The process of carcinogenesis can be broadly categorized into three distinct tumor phases: initiation, promotion and progression, i.e., metaplasia, dysplasia and anaplasia, respectively. Tumor initiation includes the transformative process by which a cell de-differentiates itself or changes from one phenotype to another to enter into hyper-proliferative and inflammatory processes. The prevailing model for cancer development is that mutations in genes for *tumor suppressors* and *oncogenes* lead to cancer. In mammals, DNA mutation cannot be avoided since their somatic cells are known to use the microhomology-mediated end joining (MMEJ) to repair double-strand breaks in DNA and this mechanism is known as an error-prone repair pathway. In MMEJ, a homology of 5–25 complementary base pairs is sufficient to align both paired strands, but mismatched ends (flaps) are usually present. MMEJ removes the extra nucleotides (flaps) where strands are joined and then ligates the strands to create an intact DNA double helix. MMEJ almost always involves at least a small deletion compared to the original sequence [26]. By extension of the causative effect of mutations in suppressor or oncogenes on tumor induction, it has also been proposed that mutations in *master genes* (controlling cell division) cause chromosome replication defects with changes in gene expression in such a way that affected cells produce too little or too much of a specific protein. If chromosomal aberrations affect the amount of one or more proteins controlling the cell cycle such as growth factors or tumor suppressors, it may result in tumor development. Excessive methylation of genes involved in cell cycle, DNA repair, and apoptosis may also lead to cancers since DNA methylation affects gene expression. Thus, different mechanisms affecting the genes involved in normal regulation of a cell or of their surrounding DNA may contribute to tumor induction or development. Cancer is essentially a disease of the regulation system of a cell that directs it to uncontrolled division and growth. The evolution of a cell toward cancer is a cumulative process that occurs on a phenotypic spectrum of increasingly disordered premalignant stages. The classic mathematical model of cell progression through tumoral stages developed by Armitage and Doll suggested that 5–8 rate-limiting events are required to generate such patterns [27].

Solid tumors whether *in vitro* or *in vivo*, are not undifferentiated masses of cells. They include necrotic regions composed of cells in quiescent state (either slowly growing or not growing at all), and regions where cells proliferate quickly. Cell’s decision to become quiescent or proliferating is thought to depend on both nutrient and oxygen availability as well as on the presence of tumor necrosis factors produced by necrotic cells that somehow inhibit further tumor growth. Mathematical models were proposed for the growth of spheroids *in vitro* [28–30] as well as of tumors *in vivo* [31].

Tumor progression involves the stroma contribution to the initiation of angiogenesis, which is the vascularization process required to sustain the energetically inefficient tumor growth under hypoxic conditions. More exactly, angiogenesis involves the proliferation and migration of endothelial cells (EC) in pre-existing vessels, while vasculogenesis involves the mobilization of bone-marrow-derived endothelial progenitor cells (EPC) into the bloodstream. In its broad sense, angiogenesis refers to the sum of angiogenesis and vasculogenesis. Once EPCs home in the tumor site, they may subsequently differentiate into ECs and contribute to the genesis of vascular structures. As far as the vascularization process is able to keep pace with demands of a growing tumor, the tumor growth rate may remain unaffected [32].

Some cancer cells acquire the ability to penetrate the walls of lymphatic and/or blood vessels, and circulate through the bloodstream to other sites and tissues in the body. At some point, they re-penetrate the vessels or walls and continue to multiply if their new hosting location is compatible with their natural environment and eventually form another clinically detectable secondary or metastatic tumor. Metastasis requires specific adhesive properties necessary for malignant cell dispersion [33]. Ultimately, incurable cancer leads to cachexia (a profound and marked state of constitutional disorder associated with a catastrophic and irreversible weight loss). The biophysical modeling of cachexia suggests that this disease state is due to a negative energy balance induced by anaerobic metabolism and excessive tumor mass at the cost of increased muscle wasting. In multiple metastatic cancers, the tumor cost could exceed patient needs to stabilize energy balance through nutrition support and bring him/her to exhaustion and accelerated demise [34].

## Consequence of tumor heterogeneity on cancer evolution and drug resistance

Whole-cancer genomes carry thousands to tens of thousands of somatic mutations, the vast majority of which probably have no biological relevance [35]. Cancer evolves dynamically as clonal expansions supersede one another driven by shifting selective pressures, mutational processes, and disrupted cancer genes. The compilation of mutational signatures from model systems exposed to known mutagens or perturbations of the DNA maintenance machinery allowed the setting up of an extensive catalogue of mutations in 30 of the most common cancer types. The procedure uncovered more than 20 signatures of processes that mutate DNA, most of them due to the AID/APOBEC family of cytidine deaminases responsible for C > T transitions on CpG dinucleotides [36]. CpG dinucleotides are hot spots of cytosine methylation whose demethylation may promote transition to thymine due to errors in the process. This process promotes the erosion of the genomic GC level; an opposite process named *kataegis* ensures an increase of the GC level by preferential incorporation of cytosine in an AT-rich context [37].

As different patterns of genomic instability have distinct genomic footprints, it is possible to interrogate sequencing and copy-number data to examine how genomic instability shapes tumor growth and evolution. Chromosome gain or loss is more likely to have functional consequences than point mutations, most of which are neutral [38]. The gains and losses of whole chromosomes or chromosome arms are well-recognized features of BC cells probably caused by mis-segregation of chromosomes during cell division [39]. The onset of large-scale chromosomal gains only starts after at least 15–20 % point mutations accumulate, but thereafter continues steadily in many tumors. However, aneuploid rearrangements occur early in tumor evolution and remain rather stable as the tumor masses clonally expand. In contrast, point mutations evolve gradually, generating extensive clonal diversity [40]. Ultimately, genome doubling can be also observed after, rather than before, the onset of chromosomal instability at later stages in disease progression [41]. Plants and animals that exhibit the process of genome doubling are generally endowed with a metabolic benefit known as *hybrid vigor* [42], which may in part explain the metabolic success of malignant cells.

**Fig. 2:**
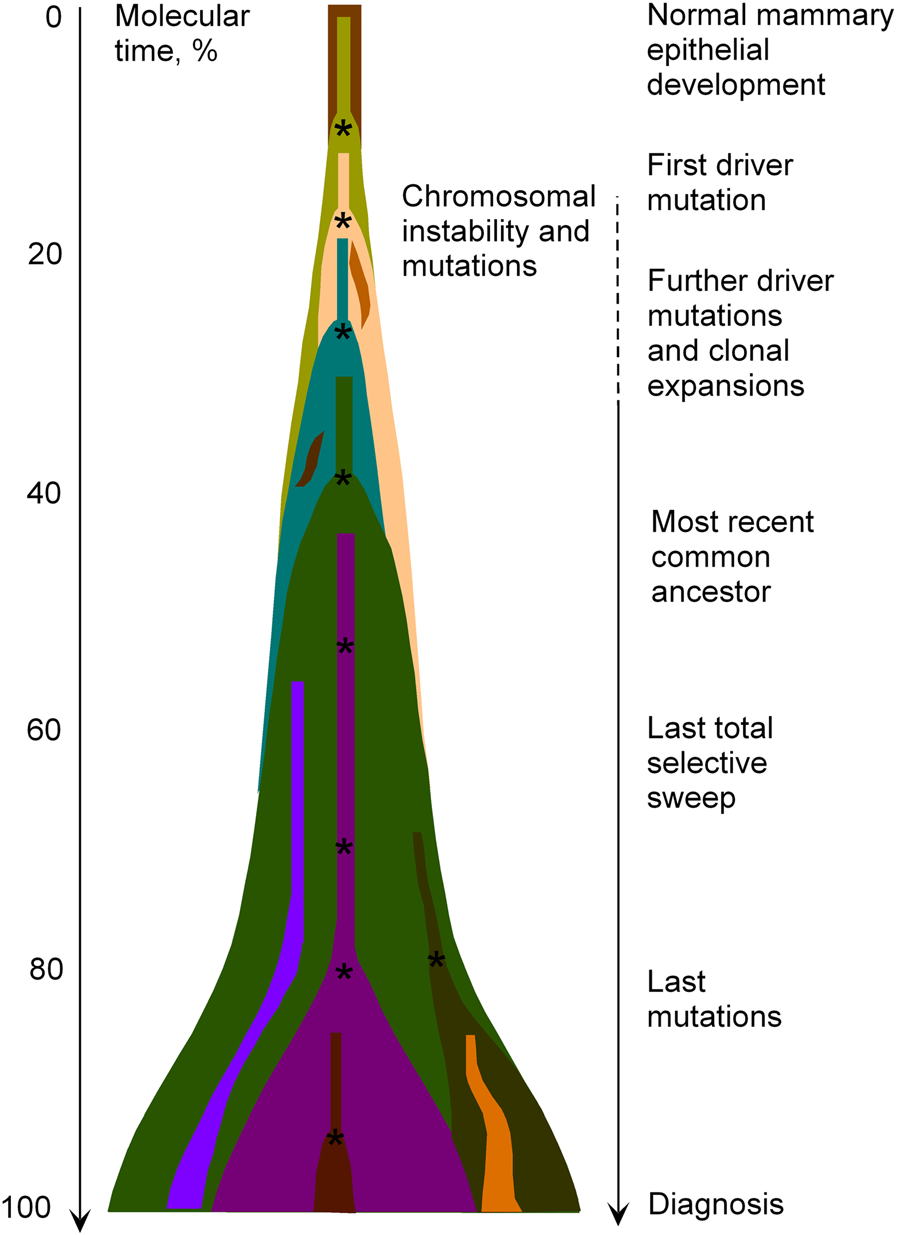
Model of tumor heterogeneity evolution over time (modified from [39])

In case of clonal sweep whereby a new clone takes over and entirely replaces the ancestral population, one observes a homogeneous cell population succeeding the previous one; this situation is an example of *linear evolution.* By contrast, if a new clone fails to outcompete its predecessor(s), a degree of heterogeneity will be observed [43], which has motivated pathologists to routinely examine multiple sections of a tumor to classify it by its highest locally observed grade [44]. When *branched tumor evolution* occurs, it results in extensive subclonal diversity [45]. It seems that in real conditions, one observes a mix of both processes since every tumor has a dominant subclonal lineage, representing more than 50 % of tumor cells (Fig. 2). Actually, there is approximately a 90 % likelihood of detecting a fully clonal mutation, a 60 % chance of detecting a mutation found in 50 % of tumor cells, and a 5 % chance of detecting a mutation in 25 % of tumor cells. Subclonal diversification is prominent, and most mutations are found in just a fraction of tumor cells. Minimal expansion of these subclones occurs until many hundreds to thousands of mutations have accumulated, implying the existence of long-lived, quiescent cell lineages capable of substantial proliferation upon acquisition of enabling genomic changes [41].

A key landmark in tumor evolution is that the most-recent common ancestor cell lineage has the full complement of somatic mutations found in all derived tumor cells. All extant cancer cells in the analyzed sample can trace a genealogy back to the initial egg cell that started the process of uncontrolled division. The most-recent common ancestor appears early in the carcinogenesis process with the consequence that much of the carcinogenesis process is devoted to subclonal diversification. One may conclude from this situation that the dominant subclone is separated from the most-recent common ancestor by many hundreds to thousands of point mutations, and that there is minimal evidence of significant clonal expansion before the accumulation of all mutations in the dominant subclone [41].

A corollary of the branched evolution of tumoral cell lineages is the high likelihood of drug resistance occurring in one of them, which indicates the need for longitudinal tumor sampling over the course of the disease and throughout treatment because the subclone that influences a disease outcome may not be detectable in a single biopsy [38]. In fact, subclones can behave in functionally distinct ways after exposure to chemotherapy and dormant resting cells surviving cytotoxic exposure can be positively selected by the treatment promoting future relapse when the patient is supposed to be disease free [46].

Increasing evidences in a variety of tumor types suggests that cells with properties of SCs are more resistant to various commonly used chemotherapeutic treatments [47]. Their persistence helps to explain the cancer recurrence following apparently successful treatment. BCSCs seem to be able to exhibit certain forms of dormancy enabling latent cancer cells to persist for years or even decades after treatment and suddenly to emerge again. Malignant cell response to therapy has been modeled by Demidenko [48] and drug resistance resulting from tumor heterogeneity can be rationalized in agreement with what is known from microbial evolution. According to the classic view of ‘survival of the fittest’, tumor cells will acquire mutations, and selection pressures will facilitate the outgrowth of some clones, but not others. Mutations provide a source of variability whose selection is applied through environmental constraints in such a way that a population explores the landscape of possible adaptation to the environmental challenges by ‘trial and error’ through its individual representatives [49]. On rare occasions, mutations provide a fitness advantage to fuel adaptive evolution and the increased mutation rate comes at the cost of increased mutational load in the genome. If beneficial mutations under strong selection occur rarely, one expects selective sweeps to drive these mutations to fixation with low resulting diversity according a linear evolution pattern. However, if these mutations occur frequently, they coexist within a population and promote its diversity according to a branched evolution pattern. By contrast, weak selection can drive diversity through the accumulation of small-effect deleterious mutations, with detrimental overall population fitness effects unless sufficient gain of a few beneficial mutations counterbalances the global figure. Exposure to drugs creates a bottleneck favoring the few clones that may randomly possess a mutation that confers resistance to the selective drug. Thus, drug treatments are expected to reduce population heterogeneity [44].

All these concepts together reinforce the notion that cancer treatment should be considered as a shift away from the *one-size-fits-all* approach, toward one in which healthcare is based on the intra- and inter-tumor heterogeneity.

## Hallmarks of cancer

The concept of a cancer hallmark tends to rationalize the complexity of neoplastic diseases in properties common to all cancer forms and that govern the transformation of normal into malignant cells. Hallmarks are acquired functional capabilities that allow cancer cells to survive, proliferate, and disseminate; these functions are acquired in different tumor types via distinct mechanisms and at various times during the course of multistep tumorigenesis. According to Hanahan and Weinberg [50], cancer hallmarks include:

(i)  
*Sustaining proliferative signaling* allowing malignant cells to stimulate their own growth. Normal cells require external growth signals (growth factors) to grow and divide. Growth factors bind cell-surface receptors, typically containing intracellular tyrosine kinase domains. The latter proceed to emit molecular signals via branched intracellular signaling pathways that regulate progression through the cell cycle as well as cell growth. By contrast, cancer cells can generate their own growth signals. Over-expressed growth factor receptors or mutated signaling protein may continuously stimulate division without the need of any growth factors in an autocrine proliferative stimulation. The numerous signaling molecules affecting cancer cells operate as nodes in interaction networks forming integrated circuits that are reprogrammed derivatives of the circuits operating in normal cells. Defects in negative feedback loops that normally operate to dampen various types of signaling to ensure homeostatic regulation of intracellular circuitry are capable of enhancing proliferative signaling [51]. Defects in the *mammalian target of rapamycin* (mTOR) signaling pathway may also promote cell proliferation [52];
(ii)  
*Growth suppressors*, i.e., resistance to paracrine inhibitory signals from their surrounding environment in the extracellular matrix and on the surfaces of neighboring cells that might otherwise stop their growth [53, 54]. These inhibitors act on the cell cycle clock, by interrupting cell division in the interphase. Ultimately, signals of growth inhibition are funneled through the retinoblastoma protein (pRB), which prevents the inappropriate transition from G1 (where cells synthesize mRNA and proteins in preparation for subsequent mitosis) to S (the cellular phase of DNA replication) [55]. If a pRB is damaged through mutation, its homing cell can start to divide uncontrollably [56];
(iii)  
*Evading cell death*, i.e., resistance to programmed cell death (apoptosis). The apoptotic machinery can be divided into sensors (IGF-1R and IL-3), which monitor the cell for abnormal behavior, and effectors (receptors of FAS and TNF-α ligands), which cause apoptosis through caspase proteases. Cell is progressively disassembled and contracts into an almost-invisible corpse that is soon consumed, both by its neighbors and by specialized phagocytic cells, upon apoptosis induction. The p53 tumor suppressor protein elicits apoptosis in response to DNA damage, and is a major protector of genome integrity. Tumors may escape apoptosis either by p53 inactivation or by increasing expression of anti-apoptotic regulators (Bcl-2, Bcl-xL) or of survival signals (Igf1/2), by down-regulating pro-apoptotic factors (Bax, Bim, Puma), or by short-circuiting the extrinsic ligand-induced death pathway. Alternatively, excessive signaling by oncoproteins such as RAS, MYC, and RAF can counteract the induction of senescence and/or apoptosis by cells [57];
(iv)  
*Enabling replicative immortality*. Normal mammalian cells have an intrinsic program, the Hayflick limit, that limits their multiplication to about 60–70 doublings that can be overcome in cancer cell by pRB and p53 tumor suppressor disabling and lead to immortalization. The clock that counts cell doubling is telomere sequences at chromosome tips by losing DNA at each cell cycle [58]. In many malignant cells, telomerase is up-regulated and telomeres are longer that in normal cells and seemingly involved in unlimited proliferation [59, 60]. However, in the human breast [61], the premalignant lesions do not express significant levels of telomerase and are marked by telomere shortening and non-clonal chromosomal aberrations suggesting that the initial involvement of p53 is disabled. Thus, the delayed telomerase activation stabilizes mutant genomes and confers the unlimited replicative capacity that cancer cells need in order to clonally expand;
(v)  
*Inducing angiogenesis*, i.e., stimulating the growth of blood vessels to supply nutrients and oxygen to tumors. The blood vessels produced within tumors by chronically activated angiogenesis are typically aberrant with tumor neovasculature marked by precocious capillary sprouting, convoluted and excessive vessel branching, distorted and enlarged vessels, erratic blood flow, micro-hemorrhaging, leakiness, as well as abnormal levels of endothelial cell proliferation and apoptosis [62]. Angiogenesis is induced by the binding of regulators, such as endothelial growth factor-A (VEGF-A) and thrombospondin-1 (TSP-1), to receptors displayed by vascular endothelial cells. The regulation of these factors can be modulated both by hypoxia and oncogene signaling [63].
(vi)  
*Activating invasion of local tissue and metastasis* or malignant cell spread to distant sites. A set of pleiotropic transcriptional factors (including Snail, Slug, Twist, and Zeb1/2) that orchestrate the epithelial-mesenchymal transition (EMT) (a means by which transformed epithelial cells can acquire the abilities to invade, to resist apoptosis, and to disseminate) and related migratory processes are expressed in various combinations in a number of malignant tumor types. They have been shown to be involved in programmed invasion [64, 65]. Cancer cells at the invasive margins of certain tumors may undergo EMT suggesting that these cancer cells are subject to micro-environmental stimuli distinct from those received by malignant cells within the tumor body [66]. The multi-step process of invasion and metastasis is presented as a succession of cellular biological changes beginning with: (i) the local invasion of surrounding stroma; (ii) the malignant cell intravasation into nearby blood and lymphatic vessels, transit of cancer cells through lymphatic and hematogenous systems; (iii) the escape of malignant cells from their lumina into parenchyma of distant tissues (extravasation); (iv) the formation of micro-metastases; and (v) the growth of micro-metastatic lesions into macroscopic tumors [67]. Concerning secondary site colonization by metastatic cells, it is worthwhile that micro-metastases that have successfully disseminated may never progress to macroscopic metastatic tumors [67, 68]. Matrix-degrading proteases are necessary to facilitate invasion into stroma, across blood vessel walls, and through normal epithelial cell layers. Metastatic cells must mimic normal cell–cell interactions, through cell–cell adhesion molecules (CAMs) and integrins. E-cadherin, which is expressed on epithelial cells [69], transmits antigrowth signals and is therefore a widely acting suppressor of invasion and metastasis that needs to be overcome by cancer cells in order to progress. The role of contextual signals in inducing an invasive growth capability (often via an EMT) implies the possibility of reversibility since cancer cells that have disseminated from a primary tumor to a distant site may no longer benefit from the favorable context of the activated stroma available in the primary tumor. In the absence of these signals, malignant cells may revert to a non-invasive state. Thus, malignant cells that have undergone an EMT during initial invasion and metastatic dissemination may pass through the reverse process of mesenchymal-epithelial transition (MET) [49]. Each type of metastatic cell needs to develop its own set of *ad hoc* solutions to the problem of thriving in a new microenvironment [70]. These adaptations might require hundreds of distinct signaling programs;
(vii)  
*Abnormal metabolic pathways.* Most cancer cells use abnormal metabolic pathways to generate energy. A hypoxic tumor microenvironment resulting from inadequate blood supply is a common feature of solid tumors. Hypoxia is a major driving force of malignant progression. It inhibits apoptosis, induces angiogenesis and the anaerobic metabolic switch, activates the EMT program, and promotes invasiveness and metastatic dissemination [71]. Glycolysis is the metabolic pathway that converts glucose to lactate. Under aerobic conditions, normal cells successively process glucose to pyruvate via glycolysis in the cytosol and to carbon dioxide in the mitochondria via oxidative phosphorylation; under anaerobic conditions, glycolysis is favored and relatively little pyruvate is dispatched to the oxygen-consuming mitochondria. Tumors generally have a high uptake of glucose relative to normal tissues. Cancer cells compensate for the ~18-fold lower efficiency of ATP production released by glycolysis relative to mitochondrial oxidative phosphorylation by up-regulating glucose transporters. Glycolytic fueling has been shown to be associated with activated oncogenes (e.g., RAS, MYC) and mutant tumor suppressors (e.g., p53) [72, 73]. The high demand for glucose together with lactate secretion, even in the presence of adequate oxygen, has been termed the Warburg effect [74]. Some tumors have been found to contain two subpopulations of cancer cells that differ in their pathways of energy supply with one subpopulation relying on the Warburg effect while the other subpopulation preferentially utilizes the lactate produced by their neighbors to generate energy through a part of the citric acid cycle [75, 76]. Glutamine may also be converted into lactate in cancer cells *in vitro* [77]. The tumor anaerobic metabolism of glucose and glutamine is a potential driver of muscle protein catabolism, as muscle is the major metabolic source of carbon for gluconeogenesis and glutamine biosynthesis. Thus, the inefficient energy tumor metabolism occurs at the cost of muscle loss and cachexia [34];
(viii)  
*Evading the immune system*, malignant cells appear to be invisible to immune system. Evidence suggests that the immune system operates as a significant barrier to tumor formation and progression. In genetically engineered mice that are immune-deficient, tumors arise more frequently and/or grow more rapidly than in the immune-competent controls [78, 79]. Highly immunogenic cancer cells seem to evade immune destruction by disabling challenging components of the immune system. For example, cancer cells may paralyze infiltrating CTLs and NK cells, by secreting TGF-β or other immune-suppressive factors [80, 81].
(ix)  
*Unstable DNA*. As outlined above, cells accumulate mutations and chromosomal abnormalities, which worsen as the disease progresses. Genomic defects induced by malfunctioning genes of DNA-maintenance machinery confer inability to: (i) properly detect DNA damage and activate repair machinery, (ii) repair damaged DNA, (iii) inactivate or intercept mutagenic molecules before they have damaged the DNA [82], and (iv) maintain telomeric DNA [58]. From a genetic perspective, these DNA-maintenance machinery genes behave much like tumor suppressor ones. The lack of genomic integrity surveillance induced by p53 deactivation may allow the survival of initial telomere erosion by other incipient neoplasias and attendant chromosomal breakage-fusion-bridge cycles. The deletions and amplifications of chromosomal segments induced by this process evidently promote genome mutability as well as mutation in oncogenes and tumor suppressor genes [58].
(x)  
*Inflammation*, the chronic tumor infiltration provides tumorigenic factors. Necrotic cells become bloated and explode, releasing their pro-inflammatory signals into the local tissue micro-environment in contrast to apoptotic cells that do not. As a consequence, necrotic cells can recruit inflammatory cells of the immune system [83, 84] attracted by associated necrotic debris. Incipient neoplasias as well as potentially invasive and metastatic tumors may gain an advantage by tolerating some degree of necrotic cell death in order to recruit tumor-promoting inflammatory cells that bring growth-stimulating factors to the surviving cells. Chemo-attractants recruit the pro-invasive inflammatory cells rather than producing the matrix-degrading enzymes themselves. It is the macrophages at the tumor periphery that supply matrix-degrading enzymes such as metallo-proteinases [85] and cysteine cathepsin proteases [86]. Therefore, they promote local tissue invasion by tumor cells. In addition, *tumor-associated macrophages* (TAM) also supply epidermal growth factor (EGF) to BC cells, while the cancer cells reciprocally stimulate the macrophages in such a way that their concerted interactions facilitate malignant cells intravasation into the circulatory system and metastatic dissemination [87].

In addition to malignant cells, tumors benefit from their micro-environment manipulation, which complicates the hallmark system [88]. Normal cells, which form tumor-associated stroma are active participants in tumorigenesis rather than passive bystanders; as such, these stromal cells contribute to the development and expression of certain hallmark capabilities [89, 90]. Among stromal components that are active tumor helpers, one may note three major cell types: angiogenic vascular cells (which supply growth factors promoting multiple hallmark capabilities), infiltrating immune cells (which supply mitogenic signals to cancer cells and proteolytic enzymes that release bioactive mitogenic agents from the ECM), and cancer-associated fibroblastic cells (which secrete mitogenic epithelial growth factors). Paracrine and juxtacrine mitogenic signals supplied by stromal cell types may potentially be involved in different tumor types at virtually any stage of tumorigenesis and progression, ranging from the initiation of aberrant proliferation to the development of adaptive resistance to therapies targeting such driving oncogenic signals [88].

## Molecular targets for breast cancer treatments

**Fig. 3:**
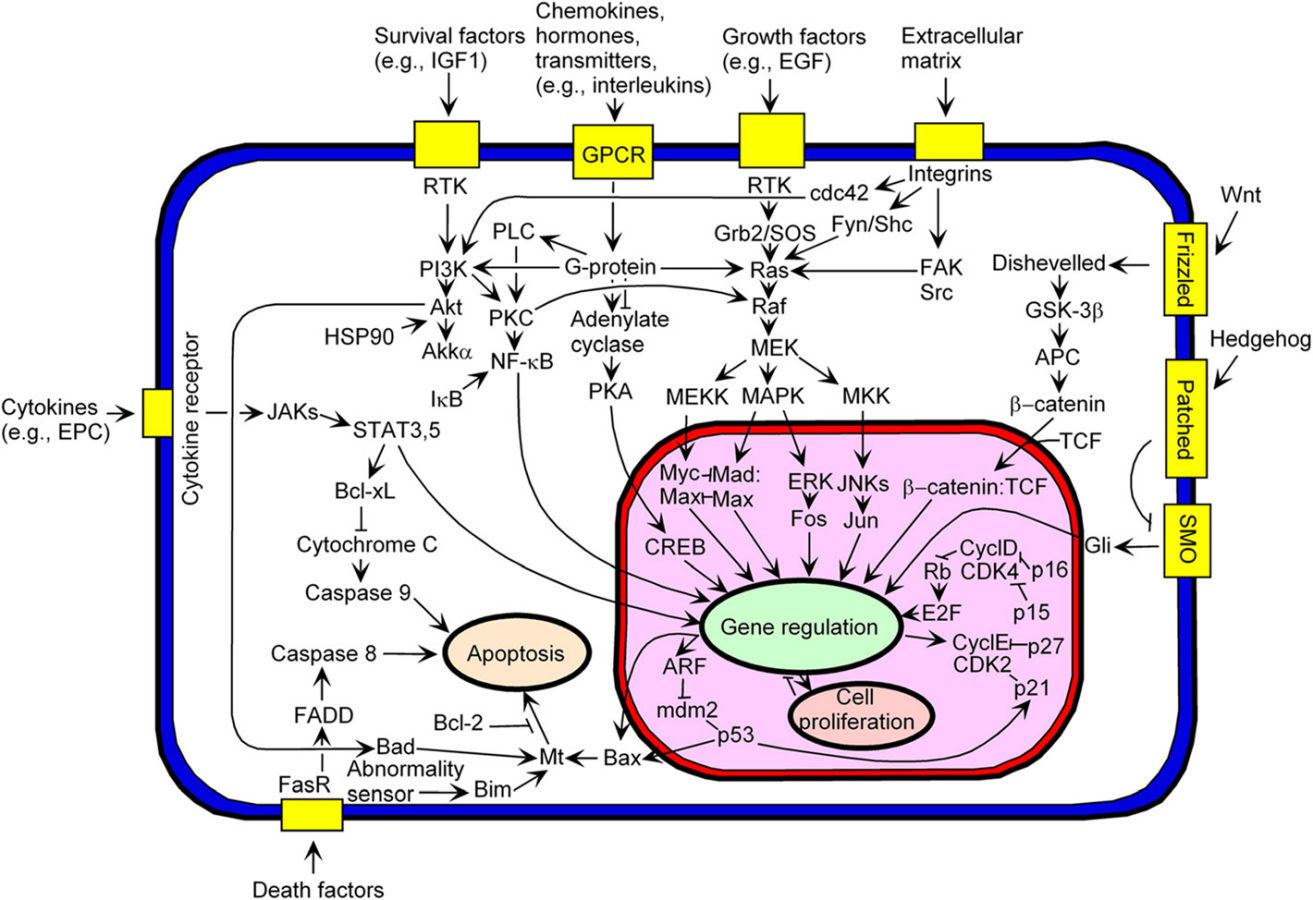
Map of key proteins in signaling pathways for the investigation of cancer therapies

As outlined above, BC resembles a Darwinian evolutionary system, with branching trajectories emerging from mutations and epigenetic changes. Such a complexity suggests that the disease control needs multi-drug cocktails. A promising strategy is to target the key phenotype features of BC cells, such as hypoxia, excessive glycolysis, angiogenesis and dedifferentiation. All these together might be targeted to transpose the hurdle of intra-tumor heterogeneity. Thus far, more than 15 different classes of target proteins have already been identified in BC along with evidence supporting drug combinations for cancer control, which deserve to be briefly listed below and are more extensively reviewed by Zardavas et al. [91]. In the state-of-the-art of modern clinical treatment, most specific drugs target proteins that belong to the signaling pathway or to trans-membrane receptors providing inputs to that pathway (Fig. 3).

The *fibroblast growth factor* (FGF) signaling pathway induces cancer cell proliferation, apoptosis evasion, facilitation of an invasive phenotype and induction of angiogenesis. There are 18 FGF ligands for four trans-membrane receptors (FGFR1–4). Activation of the pathway is associated with the consecutive activation of *phosphoinositide 3-kinase* (PI3K)/ *protein kinase B* (AKT)/mTOR, *mitogen-activated protein kinases* (MAPK), *signal transducers and activators of transcription* (STAT), and *ribosomal protein S6 kinase 2* (RSK2) signaling. The FGF pathway has been implicated in a broad range of human malignancies and promotes cancer progression in tumors driven by FGF/FGFR oncogenic mutations or amplifications responsible for tumor neo-angiogenesis and targeted treatment resistance, thereby supporting a strong rationale for anti-FGF/FGFR agent development [92, 93].

The *insulin-like growth factor* (IGF) and their receptors play pivotal roles in cellular signaling transduction and thus regulate cell growth, differentiation, apoptosis, transformation and other important physiological processes. The IGF pathway includes three trans-membrane receptors: *insulin-like growth factor 1 receptor* (IGF-1R), *insulin receptor*s (IRα and IRβ), their three ligands: IGF-I, IGF-II, insulin, and the six regulatory proteins: *insulin-like growth factor-binding proteins* (IGFBP1-6). The IGF-1R is mainly engaged in the Ras/MAPK and the PI3K/AKT pathways, and also forms cross-talks with the *epidermal growth factor receptor* (EGFR) pathway. IGF pathway is activated in more than 90 % of BC cases and is a potential target in metastatic BC. Combination of mTOR and IGF inhibitors has been shown to have a synergistic effect by inhibiting the AKT activation mediated by IGF-1R. There are many agents developed for the inhibition of IGF-1R, which are categorized into monoclonal antibodies, small molecule inhibitors and so on [94, 95].

The PI3K/AKT/mTOR signaling pathway is a hub that interconnects different oncogenic *receptor tyrosine kinases* (RTKs) with other oncogenic agents to control cell proliferation. RTKs activate: (i) the pathway (HER2 for *human epidermal growth factor receptor-2*, FGFR1, IGF-1R); the PI3K catalytic (p110α and p110β) and regulatory (p85α) subunits; the downstream PI3K effectors: AKT1 and AKT2; (ii) the PI3K activator: *Kirsten rat sarcoma homolog* (KRAS); and (iii) the negative PI3K regulators: *phosphatase and tensin homolog* (PTEN) and *inositol polyphosphate 4-phosphatase B* (INPP4B). TN cells are sensitive to simultaneous PI3K and mTOR inhibition. The mTOR kinase is a coordinator of cell growth and metabolism that lies both upstream and downstream of the PI3K pathway. mTOR activation results in the inhibition of PI3K signaling via negative feedback of some cancer cells signaling circuitry. mTOR senses and integrates diverse nutritional and environmental cues, including growth factors, energy levels, cellular stress, and amino acids [96]. It couples these signals to promote cellular growth by phosphorylating substrates that potentiate anabolic processes such as mRNA translation and lipid synthesis, or limit catabolic processes such as autophagy (autophagy is the organelle’s breakdown by a cell to supply the energy metabolism under starvation conditions). Thus, when mTOR is inhibited as is the case when applying rapamycin, the associated loss of negative feedback results in an increased activity of PI3K and its effector AKT/PKB, thereby balancing the anti-proliferative effects of mTOR inhibition induced by rapamycin [52]. Clinicians are currently faced with a wide array of clinical trials investigating a multitude of inhibitors with different mechanisms of action, being used both as single agents and in combination with other therapies [97].

The MAPK/*extracellular signal-regulated kinases* (ERK) signaling pathway is also known as the Ras-Raf-MEK-ERK pathway because it constitutes a chain of proteins that communicates a phosphorylation signal acting as an *on-* or *off-* switch from a trans-membrane protein receptor to the nuclear DNA. MAPKs are involved in directing cellular responses to a diverse array of stimuli, such as mitogens, osmotic stress, heat shock and pro-inflammatory cytokines. They regulate cell functions including proliferation, gene expression, differentiation, mitosis, cell survival, and apoptosis [98]. The MAPK/ERK1/2 signalling pathway is often dysregulated in BC and induces cellular proliferation and survival, differentiation, metastatic dissemination, as well as angiogenesis. Further characterization of the RAS-MAPK molecular regulation in malignant cells and of the acquired resistance to RAF inhibitors will facilitate development of novel combination therapies [99].

The MET (*hepatocyte growth factor receptor* - HGFR) pathway is another complex signalling network, which promote tumor progression through multiple oncogenic actions such as induction of cellular proliferation, angiogenesis, as well as invasion and metastatic dissemination through the activation of intracellular transduction systems (PI3K/AKT/mTOR, MAPK, STAT and SRC, which is a non-receptor protein tyrosine kinase) [100].

Cyclin-dependent kinases (CDKs) are a group of serine/threonine kinases that interact with specific cyclin proteins to regulate cell cycle progression. CDK bind a regulatory protein called cyclin. Without cyclin, CDK has little kinase activity and only the cyclin-CDK complex is an active kinase. CDKs are serine-threonine kinases, i.e., they phosphorylate their substrates on serines and threonines. Cancer cells override normal cell cycle checkpoints that function to halt the cell cycle as a result of DNA damage or molecular defects in the mitotic spindle. CDK inhibitors with high selectivity (particularly for both CDK4 and CDK6), in combination with patient stratification, have resulted in substantial clinical activity [101].

The Hedgehog signaling pathway has been originally described as specific to embryonic cells and is required for proper embryo development. This pathway is also found active in pluripotent BCSCs. The pathway takes its name from its polypeptide ligand, an intercellular signaling molecule called Hedgehog (Hh) found in *Drosophila*. Sonic hedgehog (SHH) is the best-studied ligand of the vertebrate pathway. When SHH reaches its target cell, it binds to the Patched-1 (PTCH1) receptor, which inhibits Smoothened (SMO), a downstream protein in the pathway that determines the fate of vertebrate limb development. Activation of the hedgehog pathway has been implicated in the BC development [102]. The Hh signaling pathway may represent a potential therapeutic target for patients with refractory pancreatic cancer. A potent Hh inhibitor can successfully inhibit tumor growth and invasiveness *in vitro* and can become a promising drug. However, in clinical trials, it has not been easy to verify the effectiveness of an Hh signaling inhibitor yet [103].

The Wnt signalling pathway is another regulator of stem cells in mammalian organisms, which is commonly dysregulated in human cancers. The Wnt name comes from the int/Wingless family from *Drosophila* that was renamed the Wnt family and int1 became Wnt1. Wnt signaling pathways are activated by the binding of a Wnt-protein ligand to a Frizzled family receptor, which passes the biological signal to the protein Dishevelled inside the cell [104]. The canonical Wnt pathway leads to regulation of gene transcription, the non-canonical planar cell polarity pathway regulates the cytoskeleton that is responsible, among other functions, for the cell shape. In embryos, Wnt controls body axis patterning, cell fate specification, cell proliferation, and cell migration and has been found to be activated in BC [105]. Drug-discovery platforms and new technologies have facilitated the discovery of agents that can alter Wnt signalling in preclinical models, thus setting the stage for clinical trials in humans [106].

## Breast cancer therapies

### Drug therapy

They are five stages (0 to IV) to describe BC. Briefly and roughly, (i) stage 0 is used to describe non-invasive BCs, (ii) stage I describes BC invading normal surrounding breast tissue, (iii) stage II describes BC invading lymph nodes, (iv) stage III describes BC invading in the lymph nodes near the breastbone and (v) the metastic stage IV that describes invasive BC spreading beyond the breast and nearby lymph nodes to other organs of the body. Chemotherapy for early stage (stage I and II) BC is not usually given as a single drug. Drugs are more commonly used in combination with one another because drug combinations have been shown to be more effective than monotherapies. Because of cytotoxic drug based therapies, the different combinations of drugs used to treat BC tend to have similar effectiveness. However, different chemotherapy combinations may be preferred for women with BC that has spread to the lymph nodes (node positive), locally advanced BC or inflammatory BC. Women with HER2+ BC may also be given *biological therapy* together with certain chemotherapy combinations.

The most common chemotherapy combinations used to treat BC are listed below:

- AC - doxorubicin (Adriamycin) and cyclophosphamide (Cytoxan, Procytox)
- AC – Taxol: doxorubicin and cyclophosphamide, followed by paclitaxel (Taxol)
- TC - docetaxel (Taxotere) and cyclophosphamide
- TAC (or DAC): docetaxel, doxorubicin and cyclophosphamide
- FAC (or CAF): cyclophosphamide (orally), doxorubicin and 5-fluorouracil (Adrucil, 5-FU)
- CEF: cyclophosphamide (orally), epirubicin (Pharmorubicin) and 5-fluorouracil
- FEC: cyclophosphamide, epirubicin and 5-fluorouracil
- FEC – T: cyclophosphamide, epirubicin and 5-fluorouracil, followed by docetaxel
- CMF – IV: cyclophosphamide (intravenous), methotrexate and 5-fluorouracil
- CMF – PO: cyclophosphamide (orally), methotrexate and 5-fluorouracil
- Taxol – FAC: paclitaxel, then followed by cyclophosphamide, doxorubicin and 5-fluorouracil
- Doxorubicin and docetaxel
- EC – GCSF: epirubicin and cyclophosphamide, with filgrastim
- Docetaxel and carboplatin (Paraplatin, Paraplatin AQ)
- Gemcitabine (Gemzar) and docetaxel
- Gemcitabine and paclitaxel
- Capecitabine (Xeloda) and docetaxel

Certain chemotherapy drugs may be used alone to treat advanced or metastatic BC. They may also be given to women who have BC that is no longer responding to other treatments. This is because single drugs have fewer side effects than drug combinations.

Drugs used to treat BC in the clinic are listed below together with their molecular targets:

- Ado-Trastuzumab Emtansine - targeting the Her2/neu receptor
- Adrucil (Fluorouracil, 5-FU) - an anti-metabolite
- Afinitor (Everolimus) - an mTOR inhibitor
- Aredia (Pamidronate Disodium) - a biophosphonate
- Arimidex (Anastrozole) - an estrogen synthesis inhibitor
- Aromasin (Exemestane) - an estrogen synthesis inhibitor
- Cisplatin - a DNA intercalator
- Clafen (Cyclophosphamide) - a DNA alkylating agent
- Doxorubicin Hydrochloride - a DNA intercalator
- Ellence (Epirubicin Hydrochloride) - a DNA intercalator
- Eribulin Mesylate - a microtubule inhibitor
- Etoposide (Vesepid, VP-16) – a topoisomerase II inhibitor
- Fareston (Toremifene) - a selective estrogene receptor modulator (SERM)
- Faslodex (Fulvestrant) - a selective estrogene receptor degrader (SERD)
- Femara (Letrozole) - an estrogen synthesis inhibitor
- Folex (Methotrexate) - an anti-metabolite and an anti-folate
- Fulvestrant - an estrogen receptor antagonist
- Gemzar (Gemcitabine Hydrochloride) - a nucleoside analog
- Herceptin (Trastuzumab) - targeting the Her2/neu receptor
- Ibrance (Palbociclib) - selective inhibitor of the cyclin-dependent kinases CDK4 and CDK6
- Ixempra (Ixabepilone) - a microtubule stabilizer
- Kadcyla (Ado-Trastuzumab Emtansine) - targeting the Her2/neu receptor
- Megace (Megestrol Acetate) - a progesterone receptor agonist
- Mitomycin (Mutamycin) - a DNA cross-linker
- Nolvadex (Tamoxifen Citrate) - an estrogen receptor antagonist
- Perjeta (Pertuzumab) - a HER2 dimerization inhibitor
- Taxol (Paclitaxel) - a microtubule stabilizer
- Taxotere (Docetaxel) - a microtubule stabilizer
- Thiotepa - a DNA alykalting agent
- Tykerb (Lapatinib Ditosylate) - a protein kinase inhibitor
- Velban (Vinblastine Sulfate) - a microtubule destabilizer
- Velsar (Vinblastine Sulfate) - a microtubule destabilizer
- Vinorelbine (Navelbine) - a microtubule destabilizer
- Xeloda (Capecitabine) - a metabolite of 5-FU
- Zoladex (Goserelin Acetate) - a gonadotropin releasing hormone superagonist

Most of the drugs listed above target tubulin and microtubules, are anti-metabolites, or target specific hormone receptors. The latter class is used in those subtypes of BC in which hormone receptors are known to be up-regulated (estrogen, progesterone, HER2).

### Hormone therapy

Hormone therapy is a systemic therapy, which inhibits the growth of hormone-sensitive tumors by blocking the body’s ability to produce hormones or by interfering with a hormone mechanism of action. This therapy might be useful as a neoadjuvant treatment, however, it is most often used as an adjuvant therapy to help in reducing the post-surgery relapse risk and also in the case of metastases. Hormone therapy is helpful for HR+ BC, but it does not help patients whose tumors are hormone receptor negative (both ER- and PR-). Several strategies have been developed to treat HR+ BC:

(i) **Ovarian shutdown or removal:** The ovaries are the main source of estrogen in premenopausal women; estrogen levels in these women can be reduced by eliminating or suppressing ovarian function, which is called ovarian ablation. Ovarian ablation can be done permanently or temporarily. In a permanent way by oophorectomy or by treatment with radiation [107]. In the temporarily way, ovarian function can be suppressed by drug therapy using *gonadotropin-releasing hormone* (GnRH) agonists. These drugs interfere with feedback regulation by the pituitary gland that stimulates ovaries to release estrogen. The data from currently published clinical trials of GnRH agonists in adjuvant settings for premenopausal women with endocrine-sensitive BC support benefit to patients [108]. Ovarian shutdown by drug therapy or surgical removal is used only in premenopausal women. Examples of ovarian shutdown drugs that have been approved by the U.S. Food and Drug Administration (FDA) are goserelin (Zoladex®) and leuprolide (Lupron®).
(ii) **Blocking estrogen production**: Aromatase is the enzyme that converts testosterone to estradiol, which is found in the body’s muscle, skin, breast and fat. Examples of aromatase inhibitors (AIs) approved by the FDA are anastrozole (Arimidex®) and letrozole (Femara®), both of which temporarily inactivate aromatase, and exemestane (Aromasin®), which permanently inactivates aromatase. Diaby et al. [109] showed that, in both early stage and advanced or metastatic BC, newer AIs have proved to be cost-effective compared to older treatments.
(iii) **Blocking estrogen’s effects**: Several types of drugs modulate estrogen receptors:

- **Selective estrogen receptor modulators or down-regulators (SERMs or SERDs)** have a competitive binding to estrogen receptors. Examples of SERMs approved by the FDA are tamoxifen (Nolvadex®), raloxifene (Evista®), and toremifene (Fareston®). Tamoxifen has been used for more than 30 years to treat HR+ BC, and can be given for 5 to 10 years after surgery to lower the likelihood of relapse. It also lowers the emergence risk of a new BC in the other breast. Because SERMs bind to estrogen receptors, they can potentially not only work as estrogen antagonists, but also as estrogen agonists according to the tissues considered. For example, raloxifene acts to prevent bone loss and to improve lipid profiles by decreasing total and LDL cholesterol, but it may also block some estrogen effects, such as those inducing breast and uterine cancers.
- **Other anti-estrogen drugs**, such as fulvestrant (Faslodex®) compete for estrogen receptor as estrogen antagonist. Upon ER binding by fulvestrant, the complex is targeted for destruction by the immune system. Fulvestrant, unlike SERMs, has no estrogen agonist effect reported.

There are three cases in which hormone therapy should be used for BC treatment: (i) adjuvant therapy for early-stage BC, (ii) treatment of advanced or metastatic BC, and (iii) neoadjuvant treatment of BC. Tamoxifen has been approved by the FDA for the adjuvant hormone treatment of premenopausal and postmenopausal women with ER+ early-stage BC, while anastrozole and letrozole have been approved in postmenopausal women. A third AI is exemestane, which has been approved as adjuvant treatment of early-stage BC in postmenopausal women who have previously received tamoxifen. Most women who received adjuvant hormone therapy are advised to take tamoxifen every day during 5 years in order to reduce the likelihood of a BC relapse [110].

A number of drugs are approved or are in clinical trials for the treatment of HR+ metastatic BCs. Investigations have shown that tamoxifen is effective in treating women with metastatic BCs; toremifene is also approved for this use. Fulvestrant can be used in postmenopausal women with metastatic ER+ BC after treatment with other anti-estrogens. Turner et al. [111] showed that the combination of palbociclib (a CDK4 and CDK6 inhibitor) and fulvestrant to treat advanced BCs has a better outcome than fulvestrant used alone. Anastrozole and letrozole can be given to postmenopausal women as initial therapy for metastatic HR+ BCs. These two drugs, as well as the aromatase inhibitor exemestane, can also be used to treat postmenopausal women with advanced BCs whose disease has worsened after tamoxifen treatment.

The use of hormone therapy to treat BC before surgery (neoadjuvant therapy) has been studied in clinical trials [110]. The goal of neoadjuvant therapy is to reduce the size of a breast tumor in order to allow breast conservation upon surgery. Data from randomized controlled trials have shown that neoadjuvant hormone therapies, in particular AIs, can be effective in reducing the size of breast tumors in postmenopausal women.

Endocrine therapy has significantly improved the outcome of patients with early- and advanced-stage HR+ BCs. However the success of hormone therapy is limited and some patients with early-stage or a metastatic stage of the disease may experience relapse or sustained disease progression. Hormonal therapy remains a controversial area with a number of unanswered questions, such as tumor resistance, patient refractoriness, optimal therapy duration, and type of complementary drugs for suitable combinations [112].

### Immunotherapy

The heterogeneous expression of tumor antigens within the primary tumor or its metastases, the modification of antigenic profile during the tumor progression, and the low levels of the antigen major histocompatibility complex proteins, as well as the low levels of other co-stimulatory proteins necessary to generate a strong immune response can explain the low immunogenicity level of tumors. Moreover, the tumor microenvironment releases immune-suppressive factors that make the antigen presentation difficult and that have a negative impact on the immune response [113]. In addition, tumors may evade immune destruction by blocking endogenous immune checkpoints that normally terminate immune responses after antigen activation. To face the low immunogenicity, the immuno-surveillance hypothesis has been refined through the concept of immune-editing where T cells from patients can be genetically engineered to express a novel T cell receptor or chimeric antigen receptor to specifically recognize a tumor-associated antigen and thereby selectively kill the corresponding tumor cells [114]. The expected benefit of immunotherapy is the specific lysis of antigen-positive cells, leaving healthy tissues intact. By using gene transfer technologies, T cells can be genetically engineered to express a unique high-affinity *T cell receptor* (TCR) or a *chimeric antigen receptor* (CAR), both of which confer novel tumor antigen specificity. An adequate number of genetically engineered T cells can therefore be produced *in vitro* for back transfer to the patient. In contrast to a TCR, which recognizes a peptide fragment of an antigen presented by an HLA molecule on the surface of target cells, a CAR molecule recognizes an intact cell surface antigen. Hence, tumor cell recognition is HLA-independent so there is no restriction in terms of patient selection. However, the requirement for the tumor-associated antigen to be a cell surface antigen excludes all mutated intracellular proteins from being targeted by CAR T cell-based therapy. The ScFv portion of the CAR molecule is generally derived from a mouse MAb. This may evoke immune responses and potential clearance of CAR-engineered T cells. To avoid this possibility, fully human CARs can be constructed [115]. Genetically engineered T cells may exert toxicity on healthy cells. Moreover, they have the potential to last for a long time in the host and even expand in number. Therefore, any adverse toxicity may worsen over time. This is a particular concern when T cells are engineered to resist the physiological signals that are exploited by many cancers to subvert tumor immune recognition and effector function. A suicide gene can be included in the genetically engineered T cells along with the CAR transgene. Cancer therapy using genetically engineered T cells is still in its infancy and the methodological diversity of TCRs and CARs preparation as well as the different preconditioning cytokine regimens will require careful optimization to be truly effective [114].

### Nanoparticle therapy

Nanoparticles are characterized by self-assembly, stability, drug encapsulation and biocompatibility as a result of their material composition. Nanoparticles are typically prepared using polyethylene glycol (PEG) as a coating material at the nanoparticle surface in order to reduce protein adsorption and complement activation [116]. The suspension of nanoparticles is very stable, and can be lyophilized. They have the potential to overcome multifactorial tumor resistance to chemotherapy due to their size between 1 and 100 nm [117]. Because aberrant morphology of their vascularization, a unique feature of solid tumors is their leaky blood vessels and defective lymphatic drainage that promotes the delivery and retention of macromolecules or nanoscale particles. Nanoparticles can be constructed at a certain size for enhanced permeability and retention effects, which is the basis for the use of nanoparticles in cancer. A careful design of nanoparticle formulation can overcome barriers posed by the tumor microenvironment and result in better treatment effectiveness. Pharmacologically active concentrations of an anticancer drug in a tumor tissue are often reached at the expense of massive body contamination with the consequence of deleterious side effects for the patient. Second-generation nanoparticles are supposed to better control deleterious side effects of drugs because of optimized intra-tumor drug delivery.

The challenge in nanoparticle technology is the optimization of their tumor targeting because of the progressive transformation of malignant cell membrane receptors due to the accumulation of genome alterations. It has been shown that 100 nm-diameter nanoparticles can passively enter tumor tissues, increasing selectivity of anticancer drug delivery at the tumor site, while markedly reducing drug accumulation and toxicity in many susceptible healthy tissues [118]. However, second-generation nanoparticles, which present surface decoration with ligands for proteins overexpressed on the surface of malignant cells are expected to substantially increase their uptake due to their increased target specificity. Unfortunately, the proper diagnosis of expressed compatible proteins on the surface of malignant cells is a bottleneck that deserves further investigation. The development of various nanoparticles with different ligands now offers a larger choice to target tumors characterized by drug resistance [119]. The use of ligands that bind specifically to malignant cell receptors may help to reduce the dose-limiting cytotoxicity of drugs and also enable drugs to bypass resistance mechanisms via cytoplasm release through endocytosis. Several clinical trials are ongoing to test the combination of: (i) monoclonal antibodies (bevacizumab, pertuzumab, trastuzumab), (ii) chemotherapy (doxorubicin, cyclophosphamide, paclitaxel, carboplatin, capecitabine, doxorubicin hydrochloride, filgrastim), and (iii) nanoparticles to improve BC treatment for early and advanced-stages (see https://clinicaltrials.gov). Another strategy that has been proposed to regulate the expression of protein targets in malignant cells has involved siRNA [120–122], but the successful target down-regulation depends on their half-life [120] and gene therapy (*clustered regularly interspaced short palindromic repeats* - CRISPR) could be necessary. However, CRISPR technology could only be envisaged if nanoparticle tumor specificity is guaranteed because permanent gene deactivation in normal cells might be another source of problems.

## High throughput technologies to assist precision therapies

The spatio-temporal organization of a developing organism requires carefully orchestrated sequences of cellular differentiation events triggered by decisions made by individual cells about their fate. Cell fate decisions are stochastic and are not reproducible at the single-cell level, but they result in highly consistent, almost deterministic patterns at the level of the whole cell population. The question of how this macroscopic order arises from a disordered microscopic behavior is reminiscent of statistical mechanics in physical systems. Cellular proliferation is punctuated by sequences of decisions that guide cell differentiation into diverse types and cell fates. These decisions are driven by chemical and mechanical signals and are highly organized in space and time, leading to well-defined macroscopic patterns, tissues and organs, in a highly reproducible manner [123]. In a developing embryo, SCs might be seen as drifting down a differentiating hill, representing the progression towards a developed organism, where they encounter branching points at which cells must decide to follow a fate over another. In culture, SCs are somehow trapped in some self-sustainable regime of pluripotent or multipotent states along the differentiation landscape. An analogy between cells in culture and statistical mechanics allows the systematic investigation of their response to controlled signals [124]. Using the standard terminology of statistical mechanics, these signals can thus be considered control parameters, and their effect can be measured in terms of a macroscopic observable, which is an output variable such as the proportion of cells within a population with a given phenotype. Cell fate decisions are associated with diverse sets of microscopic rules, defined by the genes (evaluated through genome data) and proteins involved in individual states of regulation (evaluated through transcriptome and proteome data) and by interactions (evaluated through interactome data; [125]) between them. Biological processes are considered as complex networks of interactions among numerous cell components rather than independent interactions involving only a few molecules. Because the multi-dimensional complexity of these processes involves large sample sizes, high throughput technologies are necessary to describe their time related dependencies. High throughput technologies decisively helped in developing stratified oncology. SM means analyzing large groups of cancer patients in order to predict which treatments these cancer patients are most likely to respond to. It involves looking in detail at the cancer cells and their genetic make up. Nowadays, science is able to classify cancers according to their heterogeneity and main molecular markers and the stratified oncology knowledge is progressively integrated with patient therapy to improve disease outcome considering features such as personal medical history, physiological index, molecular status of tumors, which represents the arsenal of PM tools.

### Genome sequencing

Cancers are interlinked to each other through a number of pathways, which are altered in different diseases [126]. Next generation sequencing (NGS) has been a significant technological advance for improving the understanding of malignant neoplasm because cancer is basically viewed as a genome disease. As outlined above, genome sequencing has allowed the characterization of chromosome abnormalities as gene deletion and amplification, translocation or sequence inversion as well as an epigenetic landscape. The most significant impact of next-generation sequencing on cancer genomics has been the ability to re-sequence, analyze and compare the matched tumor and normal genomes of a single patient. With the significantly reduced cost of sequencing, it is now possible to sequence multiple patient samples of a given cancer type. NGS sequencing is useful to understand the affected pathways behind cancer development. This requires a preliminary investigation to map genes that potentially lead to tumor development (oncogenes) since many mutations may occur without carcinogenic consequences. This calibration step typically involves: (i) comparison with other sequenced genomes (via dbSNP) and to other resources for variant discovery such as the 1000 Genomes Project (www.1000genomes.org), followed by (ii) comparison of remaining variant sites between the tumor and the normal genome. Another caveat of this approach is the decision whether a mutation diagnosis is a *false positive*, which tends to result from incorrect interpretation, a *false negative*, which is harder to evaluate and mainly appears as a lack of sequencing coverage or is actually correct (*true positive*). Information about the prevalence of any mutation in a cell population allows one to infer how early in the path toward cancer development that particular mutation occurred [127].

Exome is a part of the genome formed by exons, which are the protein-coding portions of genes. The whole exome sequencing information can reflect the mutations of the protein-coding region in the genome and depict the causal relationship between the mutations and phenotypes. Whole exome sequencing can achieve higher sequence depth with less raw sequence and lower cost than whole genome sequencing since exome is about 1 % of the genome size in humans. A key challenge for researches is to distinguish between driver mutations that lead to cancer development and passenger mutations, which are functionally neutral and do not contribute to tumorigenesis. A common method for identifying driver mutations is to find genes, which are recurrently mutated in large cancer samples. Initially, cancer genes such as p53, Myc, PTEN and IDH1 were recurrently discovered suggesting their role as key driver genes, but ample evidence demonstrates that pathways or subnetworks are better predictors because they reduce the complexity and diversity of driver mutations to be identified [128].

In normal cells, CpG islands preceding gene promoters are generally unmethylated, and tend to be transcriptionally active, while other individual CpG dinucleotides throughout the genome tend to be methylated. However, in cancer cells, CpG islands preceding tumor suppressor gene promoters are often hypermethylated, while CpG methylation of oncogene promoter regions and retro-sequences (retrotransposons, retrovirus) repeats is often decreased, which results in an aberrant pattern of gene expression compared to normal cells [129]. By contrast, hypomethylation of CpG dinucleotides in other parts of the genome leads to chromosome instability due to mechanisms such as loss of imprinting and reactivation of transposable elements. Thus, as a result of DNA methyltransferase (DNMTs) disruption, mitotic recombination and chromosome rearrangement can be promoted by a defective methylation pattern, which can ultimately end up in aneuploidy when the chromosomes fail to separate properly during mitosis. Methodological progress for high coverage and single base resolution profiling of the mammalian methylome in small numbers of cells through NGS has enabled deep analysis between cancer and epigenetic dysregulation [130].

According to the landscape just described, the Memorial Sloan Kettering Cancer Center has created a facility of PM called MSK-IMPACT, which routinely uses breakthrough technologies such as genomic screening approach and hybridization capture-based next-generation sequencing for solid tumor diagnostics [131]. A key question is what tumor sequencing might reveal; it is not yet clear whether cancer somatic alterations identified are recurrently affecting specific genes and to what extend a treatment may rely on mutation landscape description for a particular patient [127]. It has become clear that mutational and copy-number status alone are not highly predictive of drug response, hence there is an urgent need for improved *in silico* predictors of drug sensitivity [132]. In that respect, transcriptome profiling is synonymous with *what you see is what you get* and offers a benefit for the application of PM in real cases [133, 134].

### Transcriptome profiling

High-throughput RNA sequencing has vastly expanded the scope of genomic investigations. Most BC patients treated with adjuvant chemotherapy do not get any tangible survival benefit, yet are still exposed to the toxicity of the therapy. There is an urgent need for: (i) a precision diagnosis that would be positively correlated with an efficient therapeutics and (ii) predictive markers for patient’s response to chemotherapy being positively correlated with clinical outcome expectation. Since the oncogenesis process involves the dysregulation of several cellular pathways including cell cycle, growth, survival and apoptosis, high throughput transcriptome profiling provides a powerful tool to identify suitable disease markers and to establish a BC prognosis.

#### Microarray

Comprehensive gene expression profiling by microarrays enabled the study of thousands of genes in tens of samples and various gene clusters were correlated with distinct tumor phenotypes suggesting that tumor grades are associated with distinct gene expression signatures [135]. However, microarrays have two major shortcomings: They are limited to known genes and they have limited sensitivity as well as dynamic range. In addition, a number of clinical studies have often correlated alterations in the expression of individual genes with disease outcome according to contradicting results. Some important claims about markers for diagnosis and prognosis have been unreliable and only weakly reproducible or not reproducible at all and the process of development seems slow and inefficient [136]. In fact, most of molecular predictors were generated using a mix of molecularly heterogeneous tumors. Since oncogenic events are different across molecular classes, optimal predictors should be set up in each molecular class. Unfortunately, even under these conditions, comparisons of gene sets derived from various studies show little overlap. This may be due to the different types of arrays used, sample quality and defined parameters used for data interpretation. Oligonucleotide arrays have an additional step of target RNA amplification via *in vitro* transcription, leading to the loss of a linear relationship between the samples studied. In addition, a second loss of linearity occurs during the detection of the hybridized cDNA. Microarray technology is susceptible to a number of potential errors not just at the time of sampling, preprocessing and processing, but also at the time of data calibration and analysis [135]. Comparisons of gene lists derived from genetic assays that have been currently licensed for commercial use show limited or zero overlap between signatures. The reasons for this disparity have been attributed to differences in the groups of patients analyzed (ER status, tumor grade, stage, etc.), in sample preparation (bulk, microdissected, etc.), in microarray platforms (high or low coverage of the human genome) and in the statistical methods used (supervised or unsupervised methods, gene selection, construction of the classifiers, etc.). The lack of standardization in the setup methodology of these testes has resulted in poor prognostic reproducibility [137, 138].

#### RNA-seq

The RNA content and RNA make-up of a cell depend very much on its developmental stage and on its type. Embryonic SCs express fewer genes with an average of 22,000 mRNA molecules per cell compared to embryonic fibroblasts whose average number of mRNA molecules is 505,000 per cell, suggesting that the latter cell type contained a ~20-fold more mRNA. The same difference is observed for ribosomal RNA (small subunit), suggesting that embryonic SCs contain overall less RNA than embryonic fibroblasts, on the order of a 5.5-fold difference in total RNA [139]. A typical mammalian cell contains 10–30 pg total RNA, with 10 pg corresponding to ~103,000 mRNA molecules, on average. The majority of RNA molecules are tRNAs and rRNAs. mRNA accounts for only 1–5 % of the total cellular RNA although the actual amount depends on the cell type and physiological state. Approximately 360,000 mRNA molecules are present in a single mammalian cell, and are made up of approximately 12,000 different transcripts with a typical length of around 2 kb. Some mRNAs comprise 3 % of the mRNA pool whereas others account for less than 0.1 %. These rare or low-abundance mRNAs may have a copy number of only 5–15 molecules per cell [140].

**Fig. 4:**
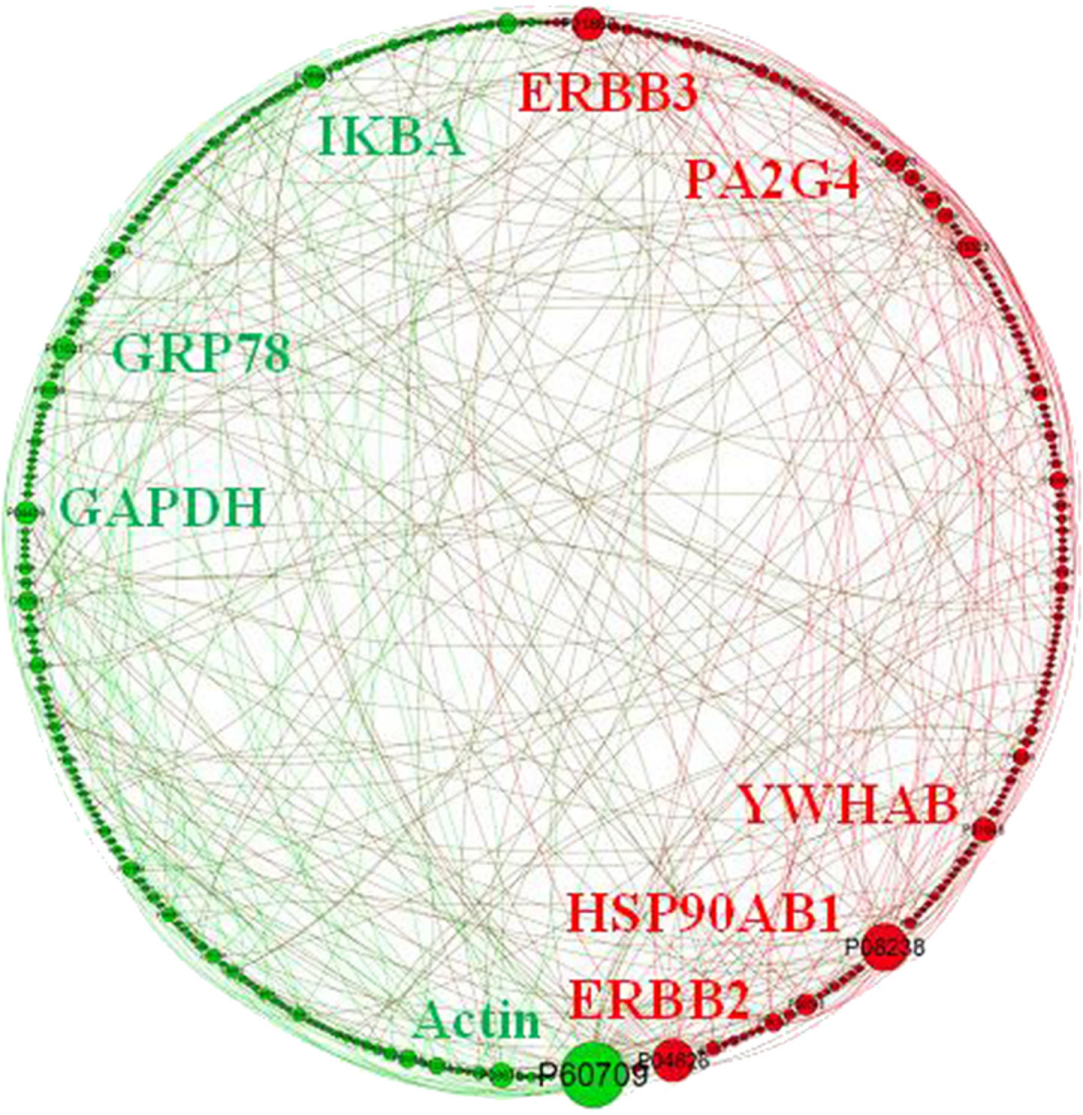
Sub-network of differentially expressed genes obtained by subtracting the RNA-seq of MCF10A (control) from that of MDA-MB-231 (Triple-Negative), represented in a circular layout. Nodes represent genes while links represent interaction between genes. Size nodes indicate connectivity and color represent an expression pattern between malignant versus non-malignant breast cell line (green: down-regulated, red: up-regulated). Gephi was used for network visualization (from [131])

These considerations are important when comparing transcriptomes of different cell types and show the need for a normalization not only for read number according to coding sequence size [141], but also for differences of mRNA per experiment that result from variation of mRNA content per cell type or sequencing coverage [142]. Typically, the transcriptome of a cell line or tumor sample is sequenced, normalized and compared to a sample of normal tissue by subtraction of relative read count per gene. Genes with a statistically significant expression level at P < 0.001 are considered up-regulated or down-regulated according to the normal sample used as a control [133] (Fig. 4). RNA-seq gives a measure of gene expression that is much more precise and reproducible than that obtained by microarrays and in agreement with qRT-PCR, which allows the extension of basic research (RNA-seq) to clinical application by qRT-PCR or Ampliseq. Ampliseq refers to NGS sequencing of amplicons from a DNA or mRNA sample [143]; this method allows the measure of relative expression levels of a predetermined pool of chosen genes (typically ~400) with the aim of obtaining a sample signature.

Micro RNA (miRNA) constitutes another layer of gene regulation (that is referred to as the *regulome* together with transcription factors) whose evaluation is also accessible through RNA sequencing; the function of these small non-coding RNA molecules (~22 nucleotides) is the post-transcriptional regulation of gene expression by mRNA silencing [144]. The expression of miRNA is highly specific of tissues and developmental stages, and the functions of miRNAs have been appreciated in various fundamental biological processes such as cell death, cell proliferation, and stem cell division. miRNAs can act as oncogenes and tumor suppressor genes. The overall miRNA expression tends to be down regulated and miRNA level is lower in poorly differentiated breast tumors with respect to well differentiated ones. Twenty-nine miRNAs were reported to be differentially expressed in BC versus normal tissues as well as miR-143, miR-145, miR-16, and let-7a-1 in MCF-7 and T47-D cell lines (see refs in [145]). Mechanisms for aberrant miRNAs expression may occur because of genomic alterations such as insertion or deletion since 72.8 % of miRNA genes were shown to be located in regions that exhibit DNA copy number abnormalities in BC [146]. In addition to copy number alteration, aberrant DNA methylation and demethylation as well as chromatin remodeling may also account for the frequent miRNA dysregulation in BC. miRNAs are intertwined with cellular pathways, and regulated by oncoproteins and tumor suppressors such as ErbB2, Akt, NF-κB, Myc, Ras, pTEN, p53, and Rb. Incorporation of miRNA regulation into current models of molecular cancer pathogenesis is essential to achieve a complete understanding of BC development. miRNAs with pro-proliferative and anti-apoptotic activity would likely promote oncogenesis and thus may be over-expressed in cancer cells. Likewise, miRNAs with anti-proliferative and pro-apoptotic activity are likely to function as tumor suppressor genes and thus may be under-expressed in cancer cells [147].

### Proteome profiling

Proteomes carry biological information that is not accessible by genomics or transcriptomics. In humans, the proteome size is 19,629 as annotated in Swiss-Prot; signatures for ~80 % were detected by mass-spectrometry (https://www.proteomicsdb.org/). Proteome coverage rapidly saturates at approximately 16,000 – 17,000 proteins, which is similar to transcriptome coverage obtained by RNA-seq, and includes a core proteome of 10,000 – 12,000 ubiquitously expressed proteins. When comparing messenger RNA-seq and proteins, clear correlations are observed in all tissues, but the correlation coefficients are moderate and somewhat poorer than those obtained for cell lines, which can be expected from the fact that tissues generally comprise a mixture of cell types including connective tissue and blood. Both mRNA and protein levels vary greatly among tissues, but the ratio of protein and mRNA levels is remarkably conserved between tissues for any given protein suggesting that the actual amount of protein in a given cell is primarily controlled by regulating mRNA levels. Knowing the protein/mRNA ratio for every protein and transcript, it is possible to predict protein abundance in any given tissue with good accuracy from the measured mRNA abundance [148].

Considering the proteome fraction dedicated to signaling, it has been shown that at least three-fourths of the proteome can be phosphorylated. In eukaryotes, phosphorylation occurs almost exclusively on Ser (84.1 %), Thr (15.5 %), and Tyr (0.4 %) residues, which represent approximately 17 % of the total amino acids in an average human protein with most Tyr kinases only activated under specific circumstances and usually stringently negatively regulated. The vast majority of phosphorylation events together use less than 20 % of cellular ATP consumed in protein phosphorylation [149].

The proteomic landscape of TN cell lines has shown that driver mutations occur frequently in regulatory proteins such as protein kinases, E3 ubiquitin ligases, and transcription factors, which alter the physiology of the cell by modulating the abundance or activity of other proteins revealing 233 hub proteins, each associated with three or more cancer census genes and “cell cycle” the only significantly enriched gene ontology (GO) term among hub proteins [150].

A full understanding of genotype-phenotype relationships in human BC requires the description of how protein interactome network is perturbed by genome alterations. In molecular biology, an interactome is the whole set of molecular interactions in a particular cell. It refers specifically to physical interactions among proteins, also known as protein-protein interactions, i.e., physical contacts established between two or more proteins as a result of biochemical events and/or biophysical forces. Here, we more particularly refer to transient interactions among proteins in the context of signaling networks, i.e., the protein pathways that connect protein receptors on the cell surface with transcription factors that (up- or down-) regulate gene expression. Evidence of protein-protein interactions represented by binary pairs can be obtained by *mammalian protein-protein interaction trap* [151], *yeast two-hybrid* (Y2H) assays [152] or supported by multiple pieces of evidence from the literature [153]. A systematic literature bias inherent to binary PPIs is that some genes are described in hundreds of publications while others have been mentioned only in a few due to the tendency to expand knowledge from already connected proteins as a consequence of socio-economic constraints. As expected from a limitation of the Y2H, the interactome obtained with this assay is depleted of interactions among proteins containing predicted transmembrane helices. Today, the human interactome lists interactions for ~17.000 proteins.

## Network modeling

A key challenge is to understand the structure and dynamics of intracellular interactions that contribute to the structure and function of a living cell. The functioning of a cell has three layers of articulation, i.e., signaling, metabolism and transcription. Roughly speaking, signaling transmits environmental signals from membrane receptors to the nucleus through a complex protein network that ends up with transcription factors activating a layer of transcription regulation involving positive and negative feedback loops that regulate gene expression. In addition to signaling and transcription feedback, gene expression also results in metabolism maintenance through enzyme fostering. These three cell activity layers form a complex network of protein-protein (PPI), protein-DNA/RNA, and protein-ligand interactions. Mathematically, network interactions are generally displayed by either a directed or undirected graph *G =* (*V, E*) with vertex (node) and edge sets *V* and *E*, respectively. An edge appears in the graph if there is a known interaction of the two partners, for example two interacting proteins in a cell, either by direct binding or by enzymatic catalysis. A node is referred to as a node of degree *k* if it is connected to other nodes by *k* edges. The connectivity level (or rate) of a network characterizes the average number of interactions (edges) per node. When, a node has a number of interactions (connections or edges) significantly larger than the average, it is called a hub. Top-5 (or 10, or more) refers to the 5 (or 10) best items in a list for a given feature under consideration.

The connectivity of a node measures its local contribution to network complexity and can be reported in terms of statistical mechanics through the concept of entropy. In thermodynamics, entropy (usually denoted by symbol *S* and referred to as the Boltzmann entropy) is a measure of the number of specific ways in which a thermodynamic system may be internally rearranged between its microstates, which is commonly understood as a measure of disorder. In statistical mechanics Boltzmann’s equation relates the entropy *S* of an ideal gas to the quantity W, which is the number of microstates corresponding to a given macrostate, i.e.

1 $$ S={k}_B \ln W $$

where *k*_*B*_ is the Boltzmann constant equal to 1.38065 × 10^−23^ J/K. For thermodynamic systems where microstates of the system may not have equal probabilities, the appropriate generalization, called the Gibbs entropy, is:

2 $$ S=-{k}_B{\displaystyle \sum {p}_i ln\kern0.5em {p}_i} $$

Here, the subscript *i* runs over all microstates and Eq. 2 reduces to Eq. 1 if the probabilities *p*_*i*_ are all equal (to *1/W*). In information theory, entropy (the so-called Shannon entropy) is the negative of the expected value of the information contained in a message received. Mathematically speaking the Shannon entropy, *H*, of a discrete random variable *X* is a measure of the amount of *uncertainty* associated with the value of *X* when only its distribution is known. So, for example, if the distribution associated with a random variable is constant (i.e. equal to some known value with probability 1), then entropy is minimal and equal to 0.

Degree-entropy (Eq. 3) is computed for a given network as:

3 $$ H=-{\displaystyle \sum_{k=1}^Np(k) \ln }p(k) $$

where *p*(*k*) represents a probability distribution on the nodes of the network, *p*(*k*) *= V*_*k*_*/V* with *V*_*k*_ the number of nodes with degree k and *V* is the total number of nodes in the network.

Mutual information (MI) is another degree-entropy derived measure that is extensively used for network characterization (Eq. 4). It provides a natural generalization of the correlation since it measures a non-linear dependency and it is able to deal with thousands of variables (genes) on a small sample number. MI measures the dependency between two variables. For discrete variables *X* and *Y*, MI is defined as:

4 $$ I\left(X,Y\right)=-{\displaystyle \sum_{x\in X,y\in Y}p\left(x,y\right) \log \frac{p\left(x,y\right)}{p(x)p(y)}} $$

where *p*(*x, y*) is the joint probability of x in *X* and y in *Y*. In terms of degree-entropy, MI can also be defined as Eq. 5:

5 $$ I\left(X,Y\right)=H(X)+H(Y)\hbox{-} H\left(X,Y\right) $$

Network entropy provides a quantitative measure of the differentiated state of a cell [154]. It has been shown that: (i) network entropy is a discriminator of pluripotent and non-pluripotent cell-types, (ii) it can further discriminate cellular states of varying degrees of multipotency within distinct lineages, (iii) it provides a more robust and general measure of a cell’s position in the global differentiation hierarchy than gene expression signatures, and (iv) it predicts a higher cellular heterogeneity in cancer stem cells compared to ordinary cancer cells. The higher entropy of pluripotent SCs compared to normal differentiated cells [132], has been seen to be due to the necessity of SCs to maintain the option of initiating the activation of a wide number of different signaling pathways associated with commitment to diverse cell fates [155]. Interestingly, network entropy of tumors as been also correlated with their aggressiveness [154, 156] establishing a correlation between malignant cell line differentiation and aggressiveness, which is consistent with the positive relationship between cell adaptiveness and totipotency. In fact, metastatic BC is characterized by an increase in the randomness of the local expression correlation patterns [157]. According to the observation that malignant cells are engaged in a fight for survival, their largest entropy compared to normal cells establishes a positive correlation with their survival success from a Darwinian perspective [158]. It has also been shown that drugs can be classified as either cytotoxic or target-specific as well as ranked according to their likelihood of controlling a tumor given its transcriptome profile using entropy as a measure [134].

The degree distribution of most biological networks can be represented by a power law *P(k) ~ k*^*–γ*^, where γ is the degree exponent and “~” indicates the proportionality between both terms. The smaller the value of *γ*, the more important the role of the hubs is in the network. For *γ* = 2, the network is characterized by a mixture of densely (hub) and scarcely connected nodes without apparent structure. In this configuration, the largest hubs are connected (edges) with a large fraction of all nodes, which ensures fast navigability of the whole network through only a few nodes or, in other words, a small *network diameter*. For 2 > *γ* >3 (scale-free), the most connected hub is in contact with only a small fraction of all nodes according to a hierarchical structures accompanied by a decrease in network navigability and an increase in network diameter. By contrast, for *γ* >3 the network structure in hub and non-hub nodes disappears and navigation from one node of the network to another results in passing through a large node number, which increases further the network diameter and brings it closer to a random configuration where nodes are connected on average through a similar edge number and whose distribution follows a Poisson distribution [159].

Most biological networks show a scale-free topology. The anisotropic node distribution in a scale-free network can be represented by several measures including the clustering coefficient and centrality [160]. The clustering coefficient can be written as *C*_*i*_ *= 2n*_*i*_*/k*(*k–1*), where n_i_ is the number of edges connecting the *k*_*i*_ neighbours of node *i* to each other. *C*_*i*_ gives the number of triangles that go through node *i*, whereas *k*_*i*_(*k*_*i*_*–1*)*/2* is the total number of triangles that could pass through node *i*, should all of node *i*’s neighbors be connected to each other. The average clustering coefficient < *C* > characterizes the overall tendency of nodes to form clusters or groups while the average clustering coefficient *C*(*k*) of all nodes with k edges is a measure of the network’s structure. The average degree < *k*>, average path length < *λ* > and average clustering coefficient < *C* > depend on the number of nodes and edges (*V* and *E*) in the network. By contrast, the *P*(*k*) and *C*(*k*) functions are independent of the network’s size and capture generic features, which allows them to be used for network classification [159].

Centrality identifies the nodes that are the most important ones in terms of graph connectivity [161]. There are several measures of centrality, but the prominent one is betweenness-centrality (Eq. 6). Given a network graph *G*(*E,V*) consisting of nodes *V* and edges *E*, the betweenness-centrality *C*_*B*_ is a measure of the centrality of a node, *v*. Typically it is the sum of the fractions of shortest paths that pass through *v* and is given by:

6 $$ {c}_B={\displaystyle \sum_{s,t\in V}\frac{\sigma \left(s,t\left|v\right.\right)}{\sigma \left(s,t\right)}} $$

where *σ*(*s,t*) is the number of shortest paths between two nodes (*s,t*) and *σ*(*s,t|v*) is the number of those paths passing through nodes other than *v*. Here, the biological system studied represents the interactome structure for a cell, i.e., the number of edges (interactions with neighbor proteins) per node (proteins in the network). The probability distribution of the events (the probability of a given number of edges per node), coupled with the information amount (the probability of a given number of edges for the node considered multiplied by its base 2 logarithm) of every event (node), forms a random variable whose average (also termed *expectation value*) is the average amount of information. Its inverse is the network entropy generated by this distribution.

Cellular networks are generally scale-free or a mixture of scale-free and hierarchical modularity as is the case of transcription regulatory network where the distribution that captures the number of different genes interacting with a transcription factor follows a power law, while the number of different transcription factors that interact with a given gene is best approximated by an exponential function [162]. In any case, cellular networks have a disproportionate number of highly connected nodes with the consequence that their path length is ultra small [163], which indicates that local perturbations in metabolite concentrations could reach the whole network very quickly. Interestingly, in protein interaction networks, highly connected nodes (hubs) avoid linking directly to each other and instead connect to proteins with only a few interactions, which warrant the disassortativity required for network stability and fast communication between clusters (modules) where proteins associated to a same function are thought to interact [164]. Modules can be detected through their clustering coefficient *C*(*k*) due to the higher local triangle density connecting cluster nodes. This indicates that nodes with only a few edges have a high *C*(*k*) and belong to highly interconnected small modules. By contrast, the highly connected hubs have a low *C*(*k*). Module identification is complicated by the fact that scale-free property and modularity are conflicting. By definition, modularity implies the existence of clear boundaries in the system. However, in a scale-free network hubs are in contact with a high fraction of nodes, which makes the existence of relatively isolated modules unlikely. Clustering and hubs coexist, which indicates that topological modules are not independent, but combine to form a hierarchical network [165]. In addition, modules are not isolated from each other; they interact and frequently overlap, which makes search for clear module boundaries nonsense and suggests that looking for hierarchical relationship between modules of different sizes is indeed the method of choice [166].

A fundamental property of a scale-free network is its high robustness level to random perturbation (node elimination), but strong vulnerability to hub elimination that makes them collapse quickly under specific attack. This property is due to the power law properties of scale-free networks where nodes with a low connection rate are much more numerous than hubs, which makes hub inactivation much less likely to occur upon random selection. When hub nodes are eliminated, the diameter of the scale-free network increases rapidly, doubling its original value if 5 % of the nodes are removed and leading to the fragmentation of module interconnections [167].

### Gene regulatory networks

Gene regulatory networks (GRN) are statistical networks inferred from gene expression data according to the hypothesis that co-expressed genes encode interacting proteins. GRN reconstruction is a daunting task due to the fact that mRNA concentrations provide only indirect information about interactions occurring between genes and their gene products. Gene expression data are multidimensional and nonlinear due to the coordination of DNA transcription, mRNA translation, protein processing as well as mRNAs and protein turnover [168, 169].

A variety of approaches have been proposed to infer GRNs such as discrete models of Boolean networks and Bayesian networks, differential equations, regression methods and linear programming, and MI (see refs in [170]). MI has been a successful framework for additional methodology refinements. In general, these approaches start by computing the pairwise MIs between all possible pairs of genes, resulting in a MI matrix. The MI matrix is then manipulated to identify the regulatory relationships. However, such a matrix contains a huge amount of information corresponding to genes that do not show any significant link with the experimental case. This problem has been typically addressed by pairwise statistical comparison using t-testing, for instance, in order to eliminate edges scoring below a significance level threshold and to keep edges between gene pairs that maximize all combinations [169, 170]. Bootstrapping has also been applied to optimize GRN tuning [169].

The relative strengths and drawbacks of computational and statistical approaches to infer GRN remain poorly understood, largely because comparative analyses usually consider only small subsets of methods, use only synthetic data, and/or fail to adopt a common measure of inference quality. Large differences of predictive accuracy exist in the method used to infer GRNs that depend on network size, topology, experiment type, and parameter settings [171]. However, the methodology is worthwhile since it may cluster genes that are potentially acting in a same functional module and ranks modules according to their relative level of expression, which adds another layer of information when interpreting results in the light of PPI networks inferred from experimental data [133, 134].

### Protein-protein interactome

Boolean networks are a promising framework for modeling signaling networks [172]. Instead of providing quantitatively precise dynamical trajectories taken by complex networks, this class of discrete systems qualitatively predicts the sequences of states accessed by these networks along their temporal evolution through binary states. This is especially convenient for signaling and regulatory circuits where activation (1) and inhibition (0) are the basic states. According to this framework, every protein evaluates the present stimulus on all its inputs. If the overall stimulus it receives at time *t* overcomes its activation threshold, the protein is activated, or stays active if it was already active; otherwise, it turns inactive or stays inactive. The dynamics of the network proceeds in discrete time steps through the simultaneous update of the states of its nodes and flows in this state space towards attractors [173, 174]. Such attractors are particular subsets of states or a single network configuration that correspond to specific protein activation patterns and can be interpreted as distinct cell phenotypes. In the model of Fumiã and Martins [172], the repertoire of cell behaviors (attractors) is determined unambiguously by the cell microenvironment and among the 62 attractors, 47 (87.4 %) correspond to apoptotic, 3 (3.1 %) to proliferative and 12 (9.5 %) to quiescent phenotypes. Interestingly, bistability was observed upon DNA damage introduction under a scenario of normal oxygenation and nutrient supply, but mitogenic signaling. Under such circumstances, around 99.35 % of the compatible initial states were attracted to the apoptotic phenotype, while a very small fraction (0.65 %) of them reached the proliferative phenotype. The other significant outcome of this modeling approach was that the monotherapies tested were ineffective to simultaneously reverse all the malignant hallmarks and seem to be additive in their effects with the consequence that a drug cocktail is necessary for cancer control or eradication.

### Relationship between disease and protein networks

The complete list of disease genes or diseasome with a phenotype effect described allowed the design of a graph where each known disorder/disease is associated to a set of genes. This experiment has shown significant functional association between cellular network modules and disorder in ~300 interactions. It was concluded that in agreement with the GRN hypothesis, genes that contribute to a common disorder: (i) show an increased tendency for their products to interact with each other through protein-protein interactions, (ii) have a tendency to be expressed together in specific tissues, (iii) tend to display high co-expression levels, (iv) exhibit synchronized expression as a group, and (v) tend to share GO terms. Together, these correlations support the hypothesis of a global functional relatedness for disease genes and their products and offer a network-based model for the diseasome. According to these conclusions, a disorder then represents the perturbation or breakdown of a specific functional module caused by variation in one or more of the components producing recognizable developmental and/or physiological abnormalities [126]. The facts that: (i) the vast majority of disease genes (~80 %) were concluded to be merely nonessential to the cell; (ii) the expression pattern of nonessential disease genes is decoupled from the overall expression pattern of all other genes, whereas essential genes have a tendency to be coupled to the rest of the cell and contribute most to the network entropy [132]; and (iii) nonessential disease genes tend to occupy functionally peripheral and topologically neutral positions in the cellular network [126] confirm that diseases genes are activated in the context of metabolism rewiring under network dysregulation and suggest that their specific control would not impair the normal cell functioning. However, in the case of cancer cells the few dysregulated genes encoding hubs may play a central role in the navigability of rewired pathways and their deactivation is expected to be critical for the disease control by disconnecting disease modules without impairing the functioning of normal cells [134]. By extension, disease hub inactivation is expected to break down attractors that are essential for cancer progression and to bring cellular activity in alternative attractors eventually ending up into apoptosis.

## Characterization of protein targets

In an *in silico* evaluation, [134] classified drugs involved in cancer therapy [175] could be separated into two general classes, i.e., (i) agents that target specific receptors such as those including angiogenesis, cell cycle, microtubule/cytoskeleton, EGFR/FGFR/HER2/IGFR signaling pathways, Ras-Raf-MEK-MAPK-ERK pathway, mTOR pathway, PI3K-AKT pathway, HDAC epigenetic agents, and HSP90s; and (ii) broad cytotoxic chemotherapeutics including nucleotide synthesis, metabolism, DNA cross-linker and multiple targets, defined as various. They found a tendency in malignant cell lines to be more sensitive, on the average, to target-specific drugs than to broadly cytotoxic ones. Cytotoxic drugs were performing poorly, on average, since their associated –log_10_(GI50) was never larger than 5.3, on average, which is considered as a rule of thumb by the state of the art of drug development as the minimal *half cell growth inhibition* (GI50) (the concentration of a drug that is needed to inhibit 50 % of cell proliferation) necessary at a 10 μM concentration to consider a candidate molecule as a potential lead compound. By contrast, target specific drugs were showing, on the average, a –log_10_(GI50) larger than the 5.3 threshold. When comparing the entropy per node of the total protein network of a cell line to its value of –log_10_(GI50) for different drugs, a clear negative correlation (r = −0.859) could be found, on average, for target- specific drugs. The negative correlation was especially convincing when considering luminal (r = −0.923) and TN cells (r = −0.725) separately. Given that target inactivation by specific drugs appeared as a more productive strategy than therapies based on cytotoxic compounds, Carels et al. [134] listed the top-5 most connected proteins encoded by up-regulated genes according to a *p*-value of 0.1 %. The subtraction of the entropy contribution of each top-5 from the total protein network entropy corresponding to the cell line under consideration gave a net entropy corresponding to that network, which meant the predicted benefit due to the target inactivation. By interpolation with the orthogonal regression line through average network entropies of TN, luminal, and control cell lines, on one hand, and patient 5-year survival [176], on the other hand, the inactivation of most top-5 targets brought the entropy back to values close to or below the entropy of the control, which meant that top-5 targets are worth considering for drug development since they potentially offer a complete 5-year survival of the patient population under consideration. However, the exact hub number that should be ideally deactivated has not been investigated.

Let us also note here that the existence of an interacting sub-network between down- and up-regulated genes indicates that the differentially expressed genes, in addition to being induced by specific cancer pathways, are interacting with each other apparently in a compensatory way [133], which further confirms the notion that oncogenesis and tumoral progression require multiple and crosstalk signaling and that a therapy driven against up-regulated genes may also affect down-regulated ones.

The result just described has shed an important light on the outcomes of therapies based on drug cocktails, which are predicted to increase the benefit expected from chemotherapy compared to treatments based on single drugs. Thus, a method is needed that enables the malignant population to be completely eliminated within a desired time-frame, negating the possibility of recurrence and promoting drug resistance. The difficulty of eradicating a mass of malignant cells is due to the nature of reaction kinetics that governs the interaction of these cells with the therapeutic agents administered. Therapeutic agents cause an exponential decay of the malignant cell population leaving a finite number of cells at the asymptotic extremity of the time of drug treatment curve as a natural consequence of the asymptotic curve that only comes into contact with the horizontal axis at a theoretically infinite time of treatment. If this situation is not taken into consideration, the necessary finite time of treatment leaves a small number of residual malignant cells that later lead to secondary malignances. A solution that has been successfully implemented by Kapoor et al. [177] has been to consider a tumor population at a value larger than it actually is, which causes a shift to increase the treatment dose or equivalently that forces the asymptotical exponential decreasing of malignant cell decay to cut the horizontal line at a finite time interval from the treatment initiation rather than at an infinite time. For this process to be efficient, all therapeutic and cell parameters must be carefully taken into account in a nonlinear model described by differential equations. The input dose of therapeutic agents was calculated according to a predefined time at which malignant cells are planned to be extinct. This calculation was made through a reverse engineering process using control strategy whose optimality has been solved by the classical method of Lagrange multipliers. The therapeutic treatment included one chemotherapeutic (cytotoxic) and two immunotherapeutic (Interleukin and Cytotoxic T-lymphocyte) agents. Such a process could be optimized to take several target-specific chemotherapeutic agents in addition to the immunotherapeutic ones. This operation would have the effect to relax the physiological constraints into which the system is forced to operate to warrant patient safety due to the inherent toxicity of chemotherapeutic cytotoxic agents. By integrating Loewe additivity and Bliss independence [178, 179], such a strategy would allow the full modeling of oncotherapeutic PM from molecular target to drug posology and to fully investigate the nature of their relationships with respect to the signaling network components.

## Challenges of precision therapy

Today, the concept of PM is defined by using terms such as *the customization of medical treatment to an individual’s genetic profile* [180]. Although an attempt to support a unique definition of PM has been published, various competing definitions using somewhat different nomenclature (e.g. “stratified\individualized” instead of personalized) persist in the literature. But all definitions share in common some form of genetic testing to identify and target specific patient profiles in order to deliver “the right drug to the right patient” and to maximize treatment effectiveness and safety [181–183].

The fast growth of genetic knowledge has allowed the shift from individual gene testing (genetics) to multiple gene evaluation (genomics). The enthusiasm for personalized medicine in oncology has been fueled by success stories of targeted therapies in a variety of tumors based on their molecular profiles [184, 185].

Pharmacogenetics is the study of inherited genetic differences in drug metabolic pathways that can affect the response of individual patients to drugs. By definition, a pharmacogenetic interaction implies that a causal genetic factor has differential effects on outcomes in treated versus untreated patients. The prior assumption is that patients should not show any positive effects without therapy application. However, tumor heterogeneity impacts on treatment regimen as combinatorial therapies are required to target different cancer cells and it makes difficult to interpret the treatment outcome. According to the World Health Organization (WHO), there are at least 18 different histological subtypes of BC, and a variety of grading and corresponding diagnostic schemes [186–188].

Although the challenges inherent to the integration of cancer, pharmacogenetics and targeted therapies into clinical practice should require evidence of benefit to the patients, an additional important parameter to consider is the cost-effectiveness for the healthcare system [188]. In this context, the PM management of cancer implies the prescription of target-specific therapeutics that is best suited for individual patients according to the type of tumor they develop. The purpose of PM strategy is to increase the efficacy of anticancer agents and to avoid toxic side effects as much as possible, which are a critical issue in clinical oncology [189]. One may consider at first glance that personal therapies can only be more expensive than standard ones, however, such a cursory assessment does not take into account the cost of administration of an ineffective treatment and the costs associated with the loss of life in terms of societal issues.

Regarding treatment costs, patients may have health care access provided through nationally funded programs according to their geographic place of residence, but the allocated resources may vary widely. Access to newer therapies and their accompanying diagnostics is often restricted as prices often exceed the thresholds used to approve new treatments. Medical insurance companies also place restrictions on diagnostic tools for cutting-edge treatments that are not supported by third-party payers. Health cost strategies vary worldwide. However, they all have a similar background of cost-driven logic. In Brazil, for instance, patients may have recourse to justice to benefit from a breakthrough treatment not normally covered by the federal health system. Access imbalances can only increase as the identification of novel targets and treatments continues. Organizations such as the Health Economic Policy and Reimbursement Committee within the European Personalised Medicine Association and the Personalised Medicine coalition have provided recommendations to address the delivery of PM across Europe and produced working documents that summarize the need to re-evaluate existing assessment and payment systems. A key challenge is to provide evidences to support the notion that PM can increase benefits to patients while lowering overall costs. On the other hand, while the cost of implementing PM may be high, one could also ask what would be the cost of not pursuing it [5].

Over the last several years, FDA (http://www.fda.gov/Drugs/InformationOnDrugs/ApprovedDrugs/default.htm) has accelerated the rate of anticancer drug approval, but only a small fraction of these new drugs found their way to a wide clinical use. FDA currently includes 155 pharmacogenetic labels, and 52 are related to oncology. Actually, nearly half of the recent cancer drug approvals (48 %) are first class, i.e., interesting drugs that use a novel mechanism of action. The better understanding of disease mechanisms and human biology has made possible to develop more effective therapeutic approaches driven by genomics and pos-genomics targets [189, 190].

Pharmacogenomic research can have an impact on how the pharmaceutical industry develops cancer drugs by identifying the genes and their isoforms involved in the interaction between a drug and the body. Bioinformatics, cheminformatics and pathway analysis developed numerous resources for pathway and network analysis, such as Biocarta, Ingenuity, KEGG and PharmaGKB, which are being used to speed up the discovery of suitable gene targets, lead compounds (potential drugs) and new molecular targets by high-throughput drug screens (HTS). Additionally, the discovery of pharmacogenomic variants improves the design of clinical trials and optimizes the drug transit through the pharmaceutical pipeline [188, 190, 191].

### Molecular and physiological effects of drugs in precision therapies

Physicians prescribe medications based on clinical evaluation or on evidence from clinical trials. To select a drug and a dosage, physicians take care of clinical factors such as weight, gender or organ function. The individual variation that may affect drug selection or dosage, such as genetic factors, is only rarely taken into consideration [188].

In one very simple scenario, a drug may act as an agonist or an antagonist for a receptor, composed of one or more proteins. At a molecular level, the metabolite can bind to the protein’s active site, which can be a ligand-binding site, a conformation-altering site or a catalytic site. Thus, the effect of a drug can then be propagated through biochemical pathways to produce cellular and systemic physiological effects. Drug metabolism can lead to the conversion of a precursor metabolite into an active drug or to the breakdown of an active into an inactive form suitable for excretion. In some cases, structurally similar molecules (e.g. a drug that is similar to a protein’s natural ligand) can bind and affect the same region of the protein and produce pharmacological effect. The absorption and distribution as well as inter-individual metabolic variation can often be explained by genetic factors [188, 192].

The most famous drug-metabolizing proteins are members of the cytochrome P450 family, which are involved in the phase I metabolism of the majority of known drugs. Polymorphisms in these genes have been involved in human drug response variation and can affect up to 25 % of all therapies [193–195].

As outlined above, tamoxifen is an anti-estrogenic drug used as adjuvant in the treatment of BC that reduces substantially the mortality due to malignant cells with estrogen receptor-α (Rα). This therapy has been the main focus of a large number of studies with emphasis on germline genotyping as a tool to improve guide treatment [196, 197]. The formation of two major primary metabolites of tamoxifen, N-desmethyl-tamoxifen and 4-hydroxy-tamoxifen, is catalyzed by CYP3A4\5 and CYP2D6, respectively. The second metabolite 4-hydroxy-N-desmethyltamoxifen (endoxifen) is genetated from N-desmethyl-tamoxifen by CYP2D6, and 4-hydroxy-tamoxifen substantially less by CYP3A4\5 [198–200]. The endoxifen and 4-hydroxy-tamoxifen are potent anti-estrogenic metabolites with a higher suppression rate of cell proliferation compared to tamoxifen, which brings out key roles of CYP2D6 and CYP3A4\5 in tamoxifen bioactivation [199]. FDA recommends CYP2D6 genotyping upon tamoxifen administration in postmenopausal and premenopausal women [197, 201] because of variations in inter-individual response due to genetic polymorphisms. For instance, different pharmacokinetic and pharmacodynamic effects were observed for various polymorphisms in the CYP encoding genes such as CYP2B6, CYP2C9, CYP2C19 and CYP3A4\5 [197]. For CYP2D6, there is a clear genetic effect partially explaining the inter-individual variability in endoxifen plasma concentration. Although the relationship between genetic polymorphisms and tamoxifen pharmacokinetics or pharmacodynamics is well understood, the variability in plasma concentration implies genotyping for tamoxifen’s clinical applicability. Different factors can contribute to the observed inter-study heterogeneity, such as differences in the quantification of tamoxifen and its metabolites, co-medications, administered dose, time on treatment, compliance, genotype comparison, tissues used for genotyping, deviation from the Hardy-Weinberg equilibrium, specifications of survival outcome, statistical power, methodology, and experimental design. In addition, a large number of studies are biased in the polymorphisms that are taken into account, which leads to potential phenotype misclassifications [197, 202].

PM is a fascinating issue however the clinical results have not been as encouraging as expected so far. This is particularly true in oncology where PM was expected to be the major field of application and where targeted therapies have not yet been able to replace classical chemotherapy [203, 204].

In general, target prioritization is a major issue and the discovery and development of target-specific therapies is still the main bottleneck. A major challenge of PM with target- specific drugs is that most responses are still transient, and tumors acquire drug resistance through genetic and non-genetic mechanisms.

Genomic and post-genomic era advances may increasingly provide assistance in difficult clinical decisions, such as those involved in BC management. The recent high-throughput technologies will facilitate pharmacogenomic progress and provide novel druggable molecules as well as support the design of future strategies aimed at BC control. Thus, substantial research investments are still needed to identify when and for whom genomic testing will be most beneficial for improved health and better oncology outcomes.

### Engineering precision therapies

The underlying goal of improving systemic treatments of BC is to evolve from a shotgun approach of treating every patient with relatively non-specific cytotoxic chemotherapy or hormonal therapy to a rational design in which patients are treated with therapies aimed at specific molecular targets. As a matter of fact, a significant proportion of BC patients are being over-treated: many patients are likely cured by locoregional therapy alone, but are enduring the side effects of unnecessary additional systemic therapies [205]. Predictors of prognosis would decrease acute and latent toxic effects and reduce treatment-associated costs. Because complex gene interactions control tumor phenotypes, traditional techniques focusing on a single or few genes had only limited success in the control of cancer disease and its prognosis. The identification of BC molecular subtypes and the development of prognostic as well as predictive molecular signatures through gene expression profiling have resulted in a better appreciation of the biological heterogeneity of BC [135].

In BC, genomic aberrations such as abnormal DNA copy number and their derived prognostic have not advanced much except for HER2; much less is understood about somatic mutations and therapy response according to survival expectation. By contrast to microarrays where expression level or copy number can only be reported for the pre-determined probe sequences tested, an added benefit of NGS is that it operates on the whole-genome scale where a complete representation of the population of DNA or RNA molecules in a sample can be queried simultaneously, which has prompted the Sweden Cancerome Analysis Network – Breast (SCAN-B) to sequence over 3,000 breast tumors to date [205]. The comparison of transcriptome profiling from malignant cell, neighboring stroma cells, and healthy cells potentially allows the identification of key protein targets involved in the malignant pathway rewiring [133]. The progressive phenotype drift of malignant cells is a complicating factor, which promotes tumor cell heterogeneity. However, it seems that key protein hubs are globally conserved in a tumor cell population, which makes sense according to the concept of common ancestor. If all malignant cells of a solid tumor derive from the same BCSC common ancestor that was successful in its pathway rewiring strategy, descendant cells should have kept such strategy even after adding new mutations. According to this notion, one may expect that a global sequencing of a heterogeneous cell population should keep detectable the common denominator of overexpressed genes since they should be up-regulated in most cells. By contrast, up-regulated genes from new mutations may not be detected as a consequence of averaging expression data by sequencing cell lines whose gene expression may be eventually in conflict. However, the astonishing progress of *laser capture microdissection* (LCM) is expected to improve the management of tumor heterogeneity. By combining LCM and linear amplification, it is already possible to draw a RNA-seq from only 1 ng, which provides a powerful tool for transcriptome analyses in the context of tumor heterogeneity [206].

Due to the advances achieved by basic research, it has become clear that even if the mutation space is very large at the genome level, the number of possible dysregulated pathways leading to cancer is much smaller, which inevitably implies phenotypic redundancy at the level of protein hub targets as they can be inferred through RNA-seq [133]. Consequently, the number of rewiring transformations from a dysregulated pathway to another must be even smaller. The considerations above suggest that post-treatment liquid biopsy should permit in the near future to follow the disease relapse and adjust adjuvant therapy accordingly and that the arsenal of target specific drugs should be sufficient to control cancer with marginal negative side effects to patients [134]. RNA-seq is still expensive, however, contextual knowledge should allow therapy design based only on a few representative protein markers that could be clinically monitored by Ampliseq at low cost.

PM implies precision therapy, which means that cytotoxic drugs should ideally be reduced to a reasonable minimum and substituted by target-specific drugs as much as possible. In this connection, nanoparticles hold great promise and can be used to administrate drugs and siRNAs. The Genomics of Drug Sensitivity in Cancer (GDSC) database (www.cancerRxgene.org) is a powerful tool for such design; it is freely available and currently contains drug sensitivity data for almost 75,000 experiments, describing response to 138 anticancer drugs across almost 700 cancer cell lines [207]; another interesting resource in that respect is the Cancer Cell Line Encyclopedia [208]. With the fast pace of modern technology development, we can make a safe prediction that at some point in a not-too-distant future, when a patient is diagnosed with cancer, it will be possible to rapidly and inexpensively sequence both malignant and normal cells through biopsy in order to inform the treatment plan. When specific oncotargets are identified, it will become theoretically possible to define a personalized drug cocktail on the basis of existing knowledge or even, on-the-fly, by *in silico* simulations (docking and molecular dynamics) of inhibitors with these oncotargets. Theoretically, this strategy is compatible with personalized medicine, in the sense, that whenever the strategy is designed, it can be, in principle, largely automated. Since the response rate to a specific chemotherapeutic drug might be relatively low in an unselected pre-treated patient population, it is a pre-requisite that the repurposing strategy includes pre-selection of those patients with a favorable molecular profile in their cancer cells, i.e., those patients with the highest likelihood of benefiting from the treatment. The strategy proposed by Carels et al. [134] differs from the traditional view of drug repurposing in expecting to find new indications for cocktail therapies that should affect essential pathways/mechanisms resulting in cancer cell death with minimal side effects for normal cells. In other words, the aim is to simultaneously maximize efficacy and minimize toxicity of a given treatment regimen. This strategy is expected to overcome intrinsic and acquired resistance, tumor heterogeneity, adaptation, and genetic instability of cancer cells.

Finally, pharmacogenetics is another PM dimension that cannot be neglected since a target specific drug can be effective for a given patient and not for another according to its particular profile of genetic polymorphism, which means that several drug alternatives must be available for a same target.

## Conclusions

The fundamental recognition that cancer is caused by the unregulated expansion of cells because of their somatic mutations has however contributed only a little to its treatment and it is not obvious that it can improve patient outcomes. A mutation-oriented view is not necessarily very productive in that respect. Indeed, mutations have indirect pleiotropic effects on the regulation of other genes. What must be considered is that cancer is a disease of cellular regulation pathways. Consequently, we propose that it is the signaling phenotype of dysregulated cancer cells that is the key feature to be addressed in order to achieve success in cancer precision therapy. The characterization of cancer-activated protein networks will guide combination therapies to optimize therapeutic effects with the consequence that a shift towards PM from the current SM approach will improve the clinical benefit to patients. However, the failure to deliver PM is often associated with the lack of specific drugs for the case under consideration. Thus, oncology is at the frontline of PM, moving into the use of molecular profile of individuals’ genomes to optimize their disease management and to avoid over- and under-treatment, which is common to traditional chemotherapy based on the SM approach, thus reducing toxicities associated with nonspecific modes of action of chemotherapy.

A key challenge associated with PM of cancer diseases is the heterogeneity of tumors and progressive phenotype drifting of their malignant cells that complicates precision diagnosis and therapies with the consequence that a single snap-shot biopsy at a single time-point may not be sufficient. Malignant cell resistance to drugs has resulted in the selection of an arsenal of cytotoxic drugs with severe side effects for patients. However, the combination of targeted therapies and the stimulation of the immune system could help in the process of malignant cell eradication. In addition, the rise of high throughput technologies for cell and molecular diagnosis at RNA/DNA and protein levels has raised hopes for precision medicine. The comparison of transcriptome profiling from malignant cells, neighbor stroma cells, and healthy cells potentially allows the identification of key hub targets involved in the malignant pathway rewiring. On-the-fly selection of target-specific drugs for disease-specific protein hubs is now theoretically feasible in the context of precision medicine. Biosensor technology is also rapidly improving and one may readily conclude that liquid biopsy will allow the non-invasive diagnosis and real-time monitoring of cancer evolution.

Despite its apparent high costs, PM must be pursued in the context of translational medicine simply due to ethical issues for patients, but also due to high cost incurred by the prescription of ineffective drug therapies in refractory patients and, finally, due to the social costs associated with cytotoxic therapies plagued by poor clinical outcomes and debilitating side effects. The European Medicines Agency guideline on anticancer drug evaluation already recommends the development of biomarker diagnostic methods early in clinical development, which makes irreversible the ongoing trend toward the PM approach of cancer [209]. Collaborative international programs with the purpose of evaluating PM approach for BC treatment, such as the umbrella study (which assesses the effect of different drugs in different molecular alterations either in one or several tumours) termed AURORA [210], have already been launched. As the era of stratified oncology moves into the era of PM, there is an urgent need for the integration of large-scale genomic and clinical data into information to serve as guidance to clinical decisions.

## Abbreviations

A: adenine
BC: breast cancer
BCSC: breast cancer stem cell
CAM: cell adhesion molecules
CAR: chimeric antigen receptor
CD: clusters of differentiation
CDK: cyclin-dependent kinases
CpG: a cytosine followed by a guanine
CTL: cytotoxic T lymphocyte
EC: endothelial cell
EM: extracellular matrix
EMT: epithelial-mesenchymal transition
EPC: endothelial progenitor cell
ER: estrogen receptor
GC: guanine plus cytosine
GI50: half cell growth inhibition
GO: gene ontology
GRN: gene regulatory networks
HER2: epidermal growth factor receptor 2
HTS: high-throughput drug screen
LCM: laser capture microdissection
MET: mesenchymal-epithelial transition
MMEJ: microhomology-mediated end joining
NGS: next generation sequencing
PM: precision medicine
PPI: protein-protein interaction
PR: progesterone receptor
SC: stem cell
T: thymine
TAM: tumor-associated macrophages
TCR: T cell receptor
TN: triple-negative
Y2H: yeast two-hybrid
